# Understanding pathogen–host interplay by expression profiles of lncRNA and mRNA in the liver of *Echinococcus multilocularis*-infected mice

**DOI:** 10.1371/journal.pntd.0010435

**Published:** 2022-05-31

**Authors:** Xiaofeng Nian, Li Li, Xusheng Ma, Xiurong Li, Wenhui Li, Nianzhang Zhang, John Asekhaen Ohiolei, Le Li, Guodong Dai, Yanhong Liu, Hongbin Yan, Baoquan Fu, Sa Xiao, Wanzhong Jia

**Affiliations:** 1 State Key Laboratory of Veterinary Etiological Biology, National Professional Laboratory for Animal Echinococcosis, Key Laboratory of Veterinary Parasitology of Gansu Province, Key Laboratory of Zoonoses of Agriculture Ministry, Lanzhou Veterinary Research Institute, CAAS, Lanzhou, Gansu, P. R. China; 2 College of Veterinary Medicine, Northwest A&F University, Yangling, Shaanxi, P. R. China; 3 State Key Laboratory of Veterinary Etiological Biology, National Foot and Mouth Diseases Reference Laboratory, Key Laboratory of Animal Virology of Ministry of Agriculture, Lanzhou Veterinary Research Institute, Chinese Academy of Agricultural Sciences, Lanzhou, Gansu, P. R. China; 4 The Instrument Centre of State Key Laboratory of Veterinary Etiological Biology, Lanzhou Veterinary Research Institute, Chinese Academy of Agricultural Sciences, Lanzhou, Gansu, P. R. China; 5 Jiangsu Co-innovation Center for Prevention and Control of Important Animal Infectious Disease, Yangzhou, Jiangsu, P. R. China; University of Liverpool, UNITED KINGDOM

## Abstract

Almost all *Echinococcus multilocularis* (*Em*) infections occur in the liver of the intermediate host, causing a lethal zoonotic helminthic disease, alveolar echinococcosis (AE). However, the long non-coding RNAs (lncRNAs) expression profiles of the host and the potential regulatory function of lncRNA during *Em* infection are poorly understood. In this study, the profiles of lncRNAs and mRNAs in the liver of mice at different time points after *Em* infection were explored by microarray. Thirty-one differentially expressed mRNAs (DEMs) and 68 differentially expressed lncRNAs (DELs) were found continuously dysregulated. These DEMs were notably enriched in “antigen processing and presentation”, “Th1 and Th2 cell differentiation” and “Th17 cell differentiation” pathways. The potential predicted function of DELs revealed that most DELs might influence Th17 cell differentiation and TGF-β/Smad pathway of host by *trans*-regulating SMAD3, STAT1, and early growth response (EGR) genes. At 30 days post-infection (dpi), up-regulated DEMs were enriched in Toll-like and RIG-I-like receptor signaling pathways, which were validated by qRT-PCR, Western blotting and downstream cytokines detection. Furthermore, flow cytometric analysis and serum levels of the corresponding cytokines confirmed the changes in cell-mediated immunity in host during *Em* infection that showed Th1 and Th17-type CD4^+^ T-cells were predominant at the early infection stage whereas Th2-type CD4^+^ T-cells were significantly higher at the middle/late stage. Collectively, our study revealed the potential regulatory functions of lncRNAs in modulating host Th cell subsets and provide novel clues in understanding the influence of *Em* infection on host innate and adaptive immune response.

## Author summary

The growth, proliferation and survival of *Echinococcus multilocularis* (*Em*) metacestode within the intermediate host’s liver depend on the complex regulation of host immune responses. Transcriptomic studies will likely help us to shed light on the possible function of regulatory RNA molecules in modulating the host immune response to benefit the parasite survival. As a newly discovered class of regulatory molecules, long non-coding RNAs (lncRNAs) have been shown to play a role in regulating host-pathogen interactions during viral, bacterial, or parasitic infections. Here, we reported the differential expression profiles of lncRNAs and mRNAs in the liver of mice after *Em* infection at different time points. The results of KEGG analysis showed that up-regulated mRNAs at 30 dpi were enriched in Toll-like and RIG-I-like receptor signaling pathways. Differentially expressed lncRNAs during the whole *Em* infection period may affect the differentiation of Th17 cells and TGF-β/Smad pathway of the host by regulating SMAD3, STAT1, and early growth response (EGR). These results provide a new dimension to better understand the immunomodulatory effects of *Em* on the intermediate host’s innate and adaptive immune responses and will aid in the design of new anti-parasitic strategies targeting the host’s non-protein-coding genome.

## Introduction

Alveolar echinococcosis (AE) is an important zoonotic infection caused by the metacestodes (larvae stage) of the tapeworm *Echinococcus multilocularis* (*Em*) [1]. Mortality can reach more than 90% in untreated or poorly treated AE patients after diagnosis within 10 to 15 years [2]. AE results in about 666,434 disability-adjusted life years (DALYs) annually which makes it a major public health threat and among the priority list of neglected tropical disease of the World Health Organization [3,4].

The metacestode of *Em* has a strong organ tropism towards the liver, thus, almost all infections occur in the liver of infected intermediate host [5]. Although humans are not directly involved in the life cycle of the parasite, they can also be infected [6]. In the infected liver, there is a continuous asexual and tumor-like proliferation of the metacestode tissue, inducing an intense local granulomatous infiltration and fibroblasts/myofibroblasts around the parasite, eventually leading to fibrosis and necrosis [7]. The liver is damaged by inflammation reaction, compression and direct invasion, which cause severe complication and hepatic dysfunction [8].

High-throughput methods such as microarray allow us to investigate the comprehensive and systemic analysis of gene expression change during the key organ injury after pathogen infection. Studies of mRNA profiles in the experimental mouse model of AE have shown the consequences of *Em* infection on liver cell metabolism, proliferation and/or death, liver fibrosis, and immune/inflammation response [9,10].

Long non-coding RNAs (lncRNAs) are the RNA transcripts longer than 200 nucleotides that do not encode protein [11]. As a newly discovered class of regulatory molecules, a growing body of literature shows that lncRNA can interact with diverse molecules, including DNA, RNAs, proteins [12] and even signaling receptors [13] to regulate numerous biological processes, such as cell cycle, differentiation, and metabolism, as well as in disease [14,15]. Many studies show that the expression profile of lncRNAs changes in the host during viral, bacterial, or parasitic infection, and that lncRNAs play an important role in regulating host–pathogen interactions, especially in immune modulation [16–19]. *Em* infection causes an immunotolerant status of the host response by modulating host immunity, which benefits long-term parasite survival, proliferation, and maturation [20–23]. This study aimed to uncover the lncRNA expression pattern in the host liver, as well as the potential function of lncRNA in modulating and orchestrating the inflammatory/immune response during *Em* infection. We explored the expression profiles of lncRNAs and mRNAs in the *Em*-infected mouse liver at different time points [2, 4, 8, 15, 30, 60, 90, and 150 days post-infection (dpi)] by microarray. At each time point, a function enrichment analysis of differentially expressed mRNAs (DEMs) was conducted to capture a preliminary and comprehensive overview of gene expression alterations in AE. By constructing lncRNA-mRNA and lncRNA-mRNA-transcription factor co-expression networks, we were able to predict the potential functions of the differentially expressed lncRNAs (DELs), providing a molecular biology basis for further understanding the roles of lncRNAs in host–*Em* interaction. Because we were interested in the immunological response during infection, we performed qRT-PCR, Western blotting, and downstream cytokine measurement to verify the effect of *Em* on two innate immune signaling pathways. Flow cytometric analysis and cytokine level measurement were used to estimate the response of T helper (Th) cells in infected liver. Our findings revealed the potential regulatory functions of lncRNAs in modulating the host Th cell subsets in AE and confirmed the activation of two innate immune signaling pathways by *Em* at 30 dpi, as well as the changes in Th1, Th2 and Th17 responses of the host during *Em* infection.

## Materials and methods

### Ethics statement

Animal experiments were conducted in compliance with the recommendations in the Guide for the Care and Use of Laboratory Animals and were approved by Lanzhou Veterinary Research Institute, Chinese Academy of Agricultural Sciences Ethics Committee (NO. LVRIAEC-2021-012).

### Mice and experimental design

The metacestodes were obtained from BALB/c mice intraperitoneally infected with protoscolices from a naturally infected plateau pika (*Ochotona curzoniae*) in Yushu, Qinghai province, China (S1 Text).

A total of 96 pathogen-free female BALB/c mice weighing around 25 g were purchased from Lanzhou University, Lanzhou, China, and divided randomly into two groups (48 mice each). One group was used as the case (CA), while the other served as the control (Con). They were housed in cages with a 12 h light/dark cycle and provided with food and water *ad libitum* during the entire experimental period. After being normally fed for two weeks, all mice in the CA group were injected intraperitoneally with 100 μl of protoscolex suspension containing about 1,000 *Em* protoscolices, while all mice in the Con group were given the same volume of sterile saline solution using the same surgical procedure. Each group was randomly divided into eight sub-groups (six mice per subgroup in separate cages) corresponding to eight autopsy time points (2, 4, 8, 15, 30, 60, 90 and 150 dpi).

### Sample collection

At 2, 4, 8, 15, 30, 60, 90 and 150 dpi, tail venous blood samples and liver samples were collected from the six animals in CA and Con groups. The blood samples were allowed to clot at room temperature for 30 min, followed by centrifugation at 3,500 rpm for 15 min at 20°C. The serum layer was collected, divided into aliquots and frozen at −80°C until use. In CA mice, samples of the liver were carefully taken from the periparasitic liver tissue (relatively close to the lesion to avoid contamination by *Em* tissue/cells). Control samples were taken from the corresponding liver lobe of Con mice. Then each liver sample was divided into two parts. One part was immediately frozen in liquid nitrogen and the other was used for flow cytometric analysis.

### RNA preparation and microarray

Four liver samples were selected from the six mice in each subgroup (CA and Con) at each autopsy time (2, 4, 8, 15, 30, 60, 90 and 150 dpi) for the detection of lncRNA and mRNA expression profiles. The Agilent Mouse lncRNA Microarray V3 (4*180 K, Design ID: 084388) that contains 43,698 probes for mouse mRNA and 87089 probes for mouse noncoding RNA was used in the assay (S1 Table) and data analysis of the 64 samples was conducted by OE Biotechnology Co., Ltd. (Shanghai, China). Total RNA was obtained using a commercially available kit (mirVana miRNA Isolation Kit, Ambion, AM1561) following the manufacturer’s instruction and quantified by NanoDrop ND-2000 (Thermo Scientific, USA). Agilent Bioanalyzer 2100 (Agilent Technologies, USA) was used to examine RNA integrity. The sample labeling, microarray hybridization and washing were done based on standard protocols according to the manufacturer. Briefly, total RNA was transcribed to double-strand cDNAs, followed by synthesis into cRNAs, and then labeled with Cyanine-3-CTP. The labeled cRNAs were hybridized onto the microarray. After washing, the scanning of the arrays was achieved by Agilent Scanner G2505C (Agilent Technologies, USA).

### Data pre-processing

Analysis of the array images to get the raw data was conducted by Agilent Feature Extraction software (version10.7.1.1, Agilent Technologies, USA). Genespring (version 14.8, Agilent Technologies, USA) was employed to finish the basic analysis with the raw data. The raw data were normalized with the quantile algorithm. Probes with at least one of two conditions flagged in “P” were chosen for further data analysis.

### DELs and DEMs analyses

LncRNA and mRNA expression levels between the CA and Con groups at each autopsy time point (2, 4, 8, 15, 30, 60, 90 and 150 dpi) were detected. Significant statistical differences were determined by one way ANOVA to identify DELs and DEMs. Then, the lncRNAs and mRNAs with values of *P* < 0.05 as well as |FC | ≥ 2, were filtrated as DELs and DEMs. The expression pattern of DELs and DEMs among groups was shown by a Hierarchical Clustering display. Additionally, according to the position of lncRNA in the genome relative to protein-encoding gene, lncRNA can be divided into four categories, including lincRNAs, antisense lncRNA, sense no exonic lncRNA and exonic lncRNAs generally (http://www.noncode.org/). Based on this, we investigated the biotypes and proportions of the identified DELs at each time point.

### Functional annotation

To provide insight into the biological functions of the differentially expressed genes, gene ontology (GO) classification (http://geneontology.org/) and the Kyoto Encyclopedia of Genes and Genomes (KEGG) enrichment analysis (https://www.kegg.jp/) were conducted. All three categories of GO term (biological process, cellular components, and molecular function) were analyzed, with a *p*-value of < 0.05 as the threshold for selecting significantly enriched functional GO terms and KEGG pathways.

### Construction of lncRNA-mRNA co-expression network

Pearson correlation coefficient (PCC) was used to describe the co-expression relationship between lncRNAs and mRNAs. For each lncRNA, we calculated the PCC value of its expression level with the expression level of each mRNA. LncRNA-mRNA pairs with |PCC value| ≥ 0.9 and *P* < 0.05 were filtered while the top five lncRNAs that had the most co-expression relationship with mRNAs were selected to construct the network, which was displayed by Cytoscape 3.7.1 (http://cytoscape.org/).

### *Cis*-and *trans*-regulating target prediction of lncRNAs and construction of TF-lncRNA-mRNA ternary network

For each lncRNA, we identified the mRNAs as “*cis*-regulated mRNAs” when: (1) the mRNAs loci are within a 100 kb window up- or downstream of the given lncRNA, (2) the PCC of lncRNA-mRNA expression is significant (*p*-value of correlation ≤ 0.05) [24]. To further understand the role of lncRNAs in AE, the transcription factor (TF) related to lncRNAs based on the cumulative hypergeometric test was used to construct a co-expression network with DELs. Each lncRNA could correlate with one or more TFs and each pair of lncRNA-TF resulted from some genes enrichment. Then, we introduced the mRNAs that were co-expressed with these lncRNAs and selected the top 10 by *p*-value ranking to construct the network of TF-lncRNA-mRNA [25–27].

### Validation by quantitative real-time PCR (qRT-PCR)

All the six liver samples in each subgroup at each autopsy time were processed and analyzed separately. Total RNA was extracted from each liver sample (about 50 mm^3^) using *mir*Vana miRNA Isolation Kit (Ambion, AM1561). Total RNA (1 mg) was transcribed to cDNA followed by qRT-PCR using PrimeScript RT reagent Kit with gDNA Eraser (Takara Bio Inc., RR047A) in a CFX96 instrument (Bio-Rad, USA). The reaction conditions for the qRT-PCR included an initial denaturation step at 95°C for 30 s, and 40 cycles of 10 s at 95°C and 1 min at 60°C. All experiments were operated in triplicate and β-actin was used for normalization. The relative expression levels were calculated using the 2^-△△CT^ method. The PCR primers used in this study were presented in S2 Table.

### Protein extraction and western blotting (WB)

The total protein from the liver (*n* = 6 in each subgroup) was extracted using a Tissues Total Protein Extraction Kit (Invent, SD001) for WB. Briefly, the proteins were transferred onto PVDF membranes (Bio-Rad, Hercules, CA, USA) and blocked with 5% skim milk in TBST (25 mM Tris-HCl, 125 mM NaCl, 0.1% Tween 20) for 1 h at 37°C. Then, IRAK4, IKKβ, MKK, JNK, NLRX1, or p-NF-κB primary antibody (Abcam, Cambridge, MA, USA) was incubated at 4°C overnight. After washing 5 times with TBST, the membranes were incubated with peroxidase-conjugated secondary antibody (Zhongshan Golden Bridge, Beijing, China) for 1 h. Here, β-actin was used as an internal control.

### Measurement of cytokines and chemokines in Serum

The serum levels of tumor necrosis factor-α (TNF-α), interferon alpha (IFN-α), interferon beta (IFN-β), interleukin-1 beta (IL-1β), interleukin-6 (IL-6), interleukin-12 (IL-12), CXCL10, CCL5, IFN-γ, IL-10, IL-4, IL-17A, IL-5 and CCL3 at 2, 4, 8, 15, 30, 60, 90 and 150 dpi were determined in six blood samples from each subgroup by the LEGENDplex Mouse Anti-Virus Response (13-plex) Panel (Biolegend, 740621) and ELISA according to the manufacturer’s instructions. The data were read on an LSRFortessa (BD Biosciences). IL-4, IL-17A, IL-5 (mouse IL-4/IL-17A/IL-5 ELISA kit, RayBiotech, ELM-IL-4, ELM-IL-17A, ELM-IL-5) and CCL3 (Mouse CCL3/MIP1 alpha AccuSignal ELISA Kit, Rockland, KOA0258) were determined by responding ELISA following the manufacturer’s instructions. The data were read on an ELISA microplate reader (Bio-Rad, USA). The OD (450nm) value minus the background of the plate absorbance was used as the final detection value.

### Flow cytometry

Single cell suspensions were prepared from fresh liver samples (2, 4, 8, 15, 30, 60, 90 and 150 dpi) of each mouse in the infected and control group (six mice per subgroup) using the MACS Liver Dissociation Kit (Miltenyi, 130-105-807) including enzyme treatments according to manufacturer’s protocol with the gentleMACS Octo Dissociator (Miltenyi Biotec, Germany). The suspension was filtered through a 70-μm cell strainer and washed in phosphate buffered saline (PBS). Red blood cells were removed with RBC Lysis Buffer (Thermo Fisher, 00–4333). The cell suspension was filtered with a 70-μm cell strainer again and washed prior to staining. Total numbers of cells were determined using TC20 cell counter (Bio-Rad, USA), and the cell concentration was adjusted to 1 × 10^6^ /ml in RPMI-1640. Single cell suspensions were prepared and incubated with 1-Methoxy-2-propylacetate (PMA, Sigma, 529117) (20 ng/ml), iomomycin (1 μg/ml) (Solarbio, 18800) and brefeldin A (10 μg/ml) (Sigma, B5936) at 37°C and 5% CO_2_ for 5 h. All samples were incubated with anti-mouse CD16/32 for Fc block for 15 min at 4°C and were then stained with anti-CD3-PB and anti-CD4-PE antibody for 30 min at 4°C. After the wash with PBS, the cells were fixed with Fixation/Permeabilization Kit (BD Biosciences, 554714) and were then stained with the following mouse-specific monoclonal antibodies for intracellular staining: anti-IFN-γ-PC7, anti- IL-4-APC, anti- IL-17A-PE, or control isotype antibody for 30 min at 4°C, in the dark. All antibodies were purchased from eBioscience (San Diego, CA, USA). Analyses were performed using the CytoFLEX LX 5L19C flow cytometer (Beckman Coulter, USA). Isotype matched antibodies were used as controls and the data were analyzed using FlowJo software (FlowJo10.8.1, Ashland, OR, USA).

### Statistical analysis

Experimental results were analyzed with a two-tailed, unpaired Student’s *t*-test using GraphPad Prism 9. The results were expressed as the mean ± SD. All results were considered statistically significant when p-values were < 0.05 (*P < 0.05, **P < 0.01, ***P < 0.001, ns: not significant).

## Results

### Confirmation of *Em* infection in BALB/c mice and identification of DEMs and DELs

**Fig 1:**
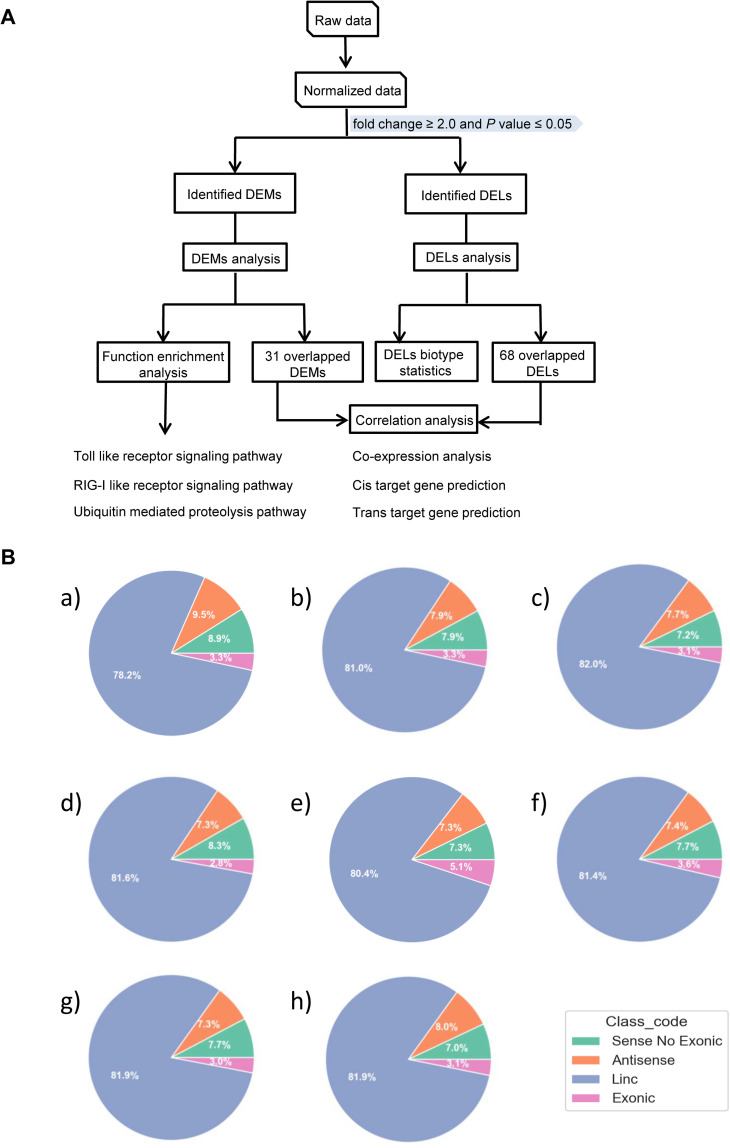
Overview of RNA microarray analysis. A. The workflow for the transcript analysis. B. Pie chart of the biotype of differentially expressed lncRNAs at 2 days post-injection (i), 4 days post-infection (ii), 8 days post-infection (iii), 15 days post-infection (iv), 30 days post-infection (v), 60 days post-infection (vi), 90 days post-infection (vii), and 150 days post-infection (vii) in the liver of *Echinococcus multilocularis*-infected mice. Antisense: which are transcribed from the antisense strand, Linc: which locate between annotated protein-coding genes, Exonic: which are transcribed from the extron of annotated coding genes and only contain one extron, Sense No Exonic: does not belong to the above three classifications.

*Em* infection was confirmed in all challenged mice by observing gross pathological lesions and detecting the lesions using the PCR amplification with *Em* specific primers (S2 Table). The infected livers were enlarged, and the surface of the liver lobes was characterized by translucent or whitish alveolar vesicles. Meanwhile, it was confirmed that all CA mice were infected successfully (S1 Fig) and all collected liver samples were not contaminated with *Em* tissue by PCR (S2 Fig). The data of mRNA and lncRNA expression profiles in mouse liver in response to *Em* at eight time points obtained by microarray in this work were deposited in NCBI’s Gene Expression Omnibus and were accessible through GEO Series accession number GSE184297 (https://www.ncbi.nlm.nih.gov/geo/query/acc.cgi?acc=GSE184297). At each time point, both of CA and Con groups were tested using four biological replicates. The workflow for the transcript analysis was shown in Fig 1A. The mRNAs and lncRNAs with an expression change of more than two-fold (FC ≥ 2) and *P* < 0.05 were selected as differentially expressed candidates. The numbers of DEMs and DELs at each time point were showed in Table 1. For DEMs, the proportions of down-regulated genes were more than 50% (51.5% to 68.3%) at all the time points except for 30 dpi (24.9%). For DELs, there were more down-regulated genes than up-regulated genes at all the time points, with proportions ranging from 58.4% to 83.8%. In general, the expression level of mRNA and lncRNA displayed a similar trend of variation. Compared with the Con groups, the expression patterns of mRNAs and lncRNAs in CA group at each time point were significantly different, see volcano (S3 and S4 Figs) and hierarchical clustering plots (S5 and S6 Figs) of visual representation. These results indicated that *Em* triggers a strong alteration of numerous mRNAs and lncRNAs expression during the infection course. Additionally, there were more DEMs and DELs at 4, 15, 60, 90, and 150 dpi than other time points, which may be indicative of a more vigorous host response at indicated time points.

**Table 1:** Numbers of differentially expressed mRNAs and lncRNAs at eight time points in mouse livers after *Echinococcus multilocularis* infection (*P* < 0.05 and fold Change ≥ 2).

Time point	Differentially expressed mRNAs (DEMs)					Differentially expressed lncRNAs (DELs)				
	up	% of all DEMs	down	% of all DEMs	total	up	% of all DELs	down	% of all DELs	total
2 dpi	230	37	395	63	625	292	21.4	1071	78.6	1363
4 dpi	724	48.5	770	51.5	1494	1182	38.9	1859	61.1	3041
8 dpi	325	42.6	438	57.4	763	491	31.6	1063	68.4	1554
15 dpi	322	31.7	695	68.3	1017	471	24.1	1487	75.9	1955
30 dpi	522	75.1	173	24.9	695	125	24.5	386	75.5	511
60 dpi	938	39.7	1427	60.3	2365	552	16.2	2848	83.8	3400
90 dpi	650	41.7	907	58.3	1557	446	17.5	2097	82.5	2543
150 dpi	1161	43.2	1525	56.8	2686	1864	41.6	2617	58.4	4481

dpi: days post infection; DEMs: differentially expressed mRNAs; DELs: differentially expressed lncRNAs

The biotypes and proportions of the identified DELs at each autopsy time point were investigated. As shown in Fig 1B, we found that biotypes of the DELs and their corresponding ratios at different time points during *Em* infection were almost the same. There were more than 78% DELs were lincRNAs. Antisense lncRNA and sense no exonic lncRNA in the DELs accounted for nearly equal proportion (7–9%), while exonic lncRNAs were fewest (~3%).

### Candidate DEMs and DEL qRT-PCR results validated the microarray data

**Fig 2:**
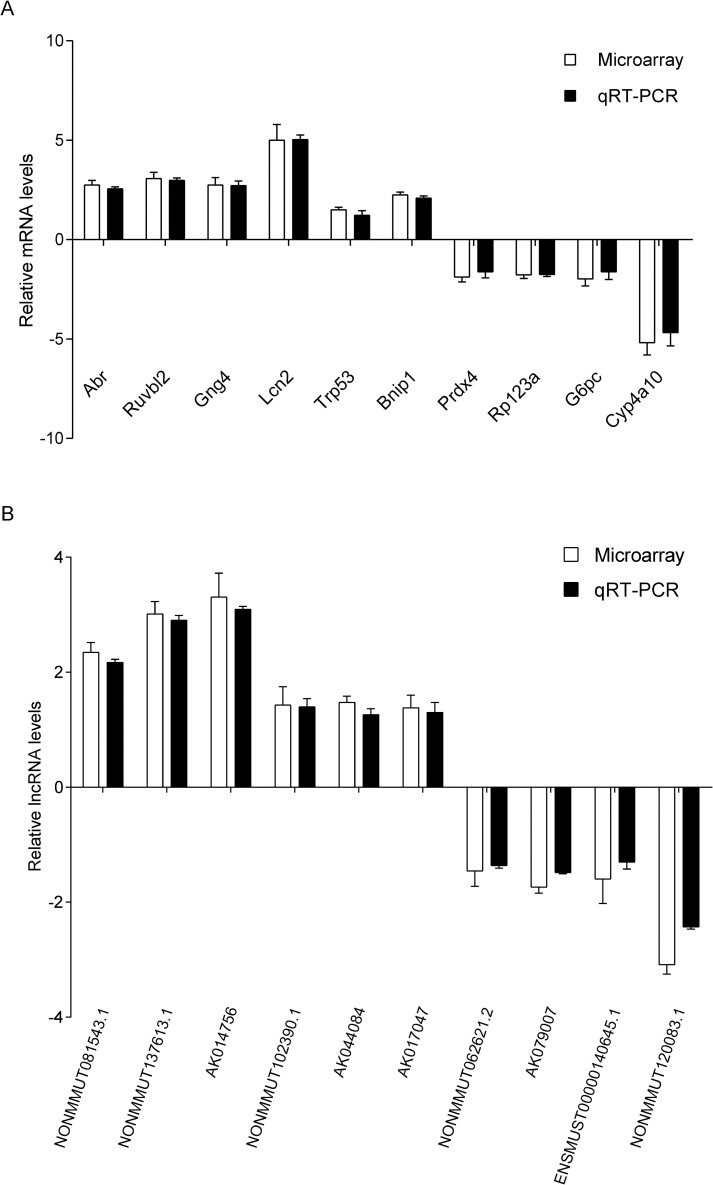
Quantitative real-time PCR (qRT-PCR) validation of 20 randomly selected transcripts at the different eight time points. A. Quantitative real-time PCR (qRT-PCR) results of 10 randomly selected differently expressed mRNA at eight time points. 10 mRNAs: Abr (up-regulated) was selected randomly at 2 days post injection; Ruvbll2 (up-regulated) was selected randomly at 4 days post-infection; Rp123a (down regulated) was selected randomly at 8 days post-infection; Gng4 (up-regulated) and Bnip1 (up-regulated) were selected randomly at 15 days post-infection; Prdx4 (down regulated) and Trp53 (up-regulated) were selected randomly at 30 days post-infection; Lcn2 (up-regulated) was selected randomly at 60 days post-infection; G6pc (down-regulated) was selected randomly at 90 days post-infection; Cyp4a10 (down-regulated) was selected randomly at 150 days post-infection. B. Quantitative real-time PCR (qRT-PCR) results of 10 randomly selected differently expressed lncRNA at eight time points. 10 lncRNAs: AK079007 (down regulated) was selected randomly at 2 days post-injection; NONMMUT137613.1 (up-regulated) was selected randomly at 4 days post-infection; ENSMUST00000140645.1 (down regulated) and AK014756 (up-regulated) were selected randomly at 8 days post-infection; NONMMUT062621.2 (down regulated) was selected randomly at 15 days post-infection; NONMMUT081543.1 (up-regulated) was selected randomly at 30 days post-infection; NONMMUT102390.1 (up-regulated) was selected randomly at 60 days post-infection; AK017047 (up-regulated) and NONMMUT120083.1 (down regulated) were selected randomly at 90 days post-infection; AK044084 (up-regulated) was selected randomly at 150 days post-infection. The data were presented as mean ± standard deviation (*n* = 6) per test in a single experiment repeated three times.

To validate the DELs/DEMs identified in mouse livers at each autopsy time point by microarray, twenty DEMs and DELs (10 each) randomly selected were verified by qRT-PCR. As shown in Fig 2, the qRT-PCR results of 10 mRNAs (six up-regulated and four down-regulated) and 10 lncRNAs (six up-regulated and four down-regulated) confirmed the microarray data showing similar trends in the up- or down-regulated mRNAs (Fig 2A) and lncRNAs (Fig 2B).

### Functional annotation of the differentially expressed mRNAs and overall gene expression changes in *Em*-infected liver

**Fig 3:**
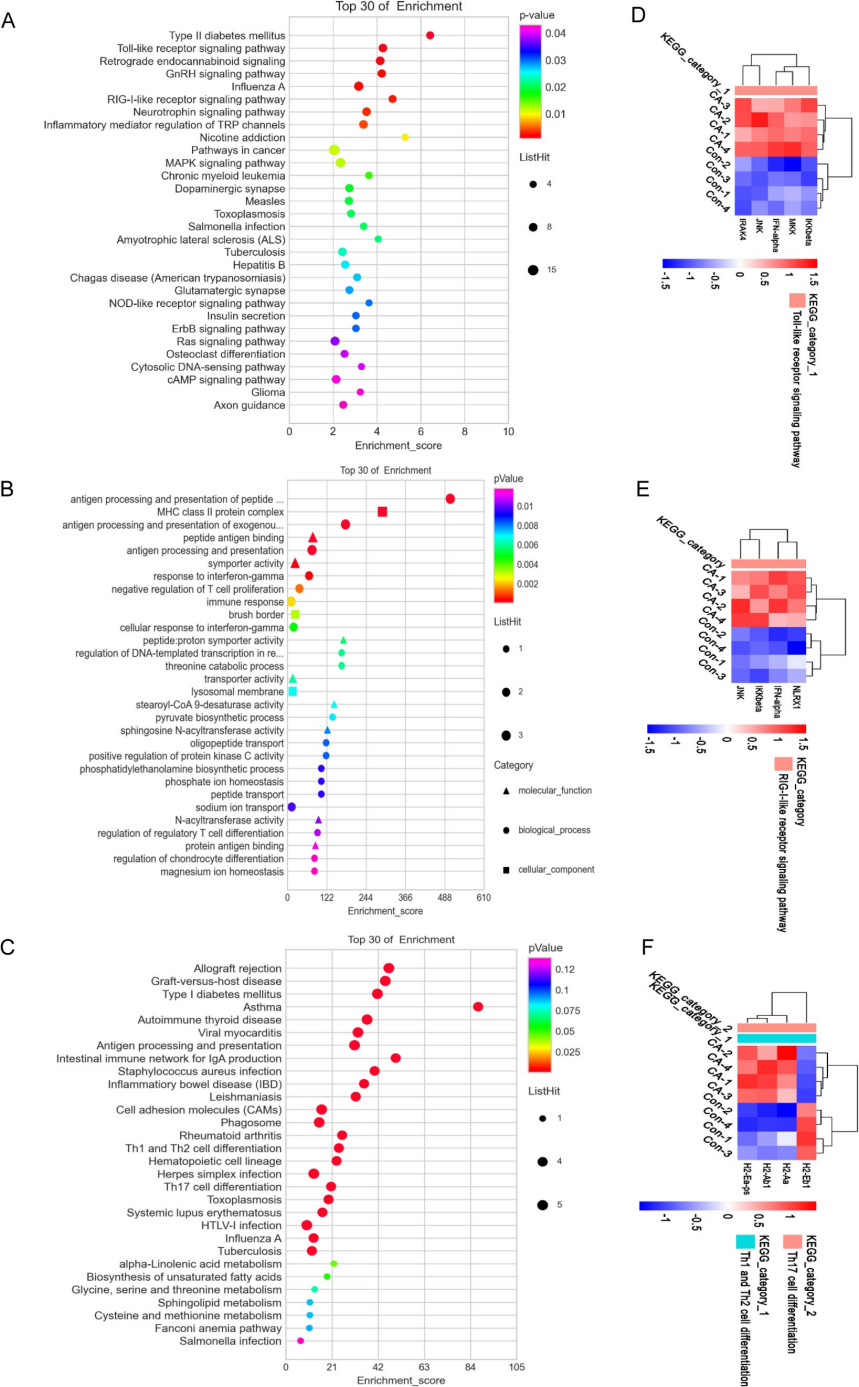
Functional annotation of the differentially expressed mRNAs and lncRNAs in *Echinococcus multilocularis*-infected mouse liver. A. KEGG analysis of up-regulated differentially expressed mRNAs at 30 dpi with top 30 enrichment scores. B. GO annotation of continuously dysregulated mRNAs at eight time points with top 30 enrichment scores including the domains of biological processes, cellular components, and molecular functions. C. KEGG analysis of continuously dysregulated mRNAs at eight time points with top 30 enrichment scores.

To have better understanding on the biological roles of differentially expressed mRNAs at each autopsy time point, the up- and down-regulated DEMs were estimated by KEGG pathway analysis. We found that Toll-like (TLRs) and RIG-I-like receptor (RLRs) signaling pathway were significantly enriched by up-regulated DEMs at 30 dpi (Fig 3A). The mRNA expression level of IRAK4, IKKβ, MKK, JNK, and IFN-α were up-regulated in the TLRs signaling pathway (S7 Fig). In the RLRs signaling pathway, the mRNA expression level of NLRX1, IKKβ, JNK, and IFN-α increased after *Em* infection (S8 Fig). The differences in the expression level of these up-regulated genes in Con and CA groups at 30 dpi were shown by the heatmaps (Fig 3D and 3E). Additionally, we found that down-regulated DEMs at 2, 4, 8, 15, 30, 60, 150 dpi were all significantly enriched in “ubiquitin-mediated proteolysis” pathway. Moreover, among these time points, there were more dysregulated genes at 15 dpi (S9 Fig). These results suggested that *Em* infection had a broad and long-lasting impact on ubiquitin-mediated proteolysis pathway in the host and 15 dpi may be the most affected time point during the infection period.

Next, we also estimated the biological roles of the genes with continued aberrant expression during the infection course. Sixty-eight DELs (down-regulated) and 31 DEMs (21 down-regulated and 10 up-regulated) were found overlapped at all the time points by Venn analysis (S3 and S4 Tables). As shown in Fig 3B, these DEMs were significantly enriched in several immune biological processes, such as “antigen processing and presentation of peptide, polysaccharide or exogenous peptide antigen via MHC class II”, “response to interferon-gamma” and “negative regulation of T cell proliferation”. Most continued aberrant DEMs were associated with cell component of “MHC class II protein complex”, “brush border” and “lysosomal membrane”. And these DEMs were enriched on some transportation-related molecular functions such as “peptide antigen binding”, “symporter activity” and “transporter activity”.

DEMs with persistent changes were analyzed by the KEGG database. As shown in Fig 3C, they were mainly involved in “antigen processing and presentation”, “intestinal immune network for IgA production”, “Th1 and Th2 cell differentiation”, and “Th17 cell differentiation” pathway, suggesting that these signaling pathways were continuously active throughout the infection and that *Em* had enduring interactions with these pathways. Here, the expression levels of DEMs involved in Th1, Th2 and Th17 cell differentiation were shown in the heatmap in Fig 3F.

### Construction of co-expression network between overlapped DELs and DEMs

**Fig 4:**
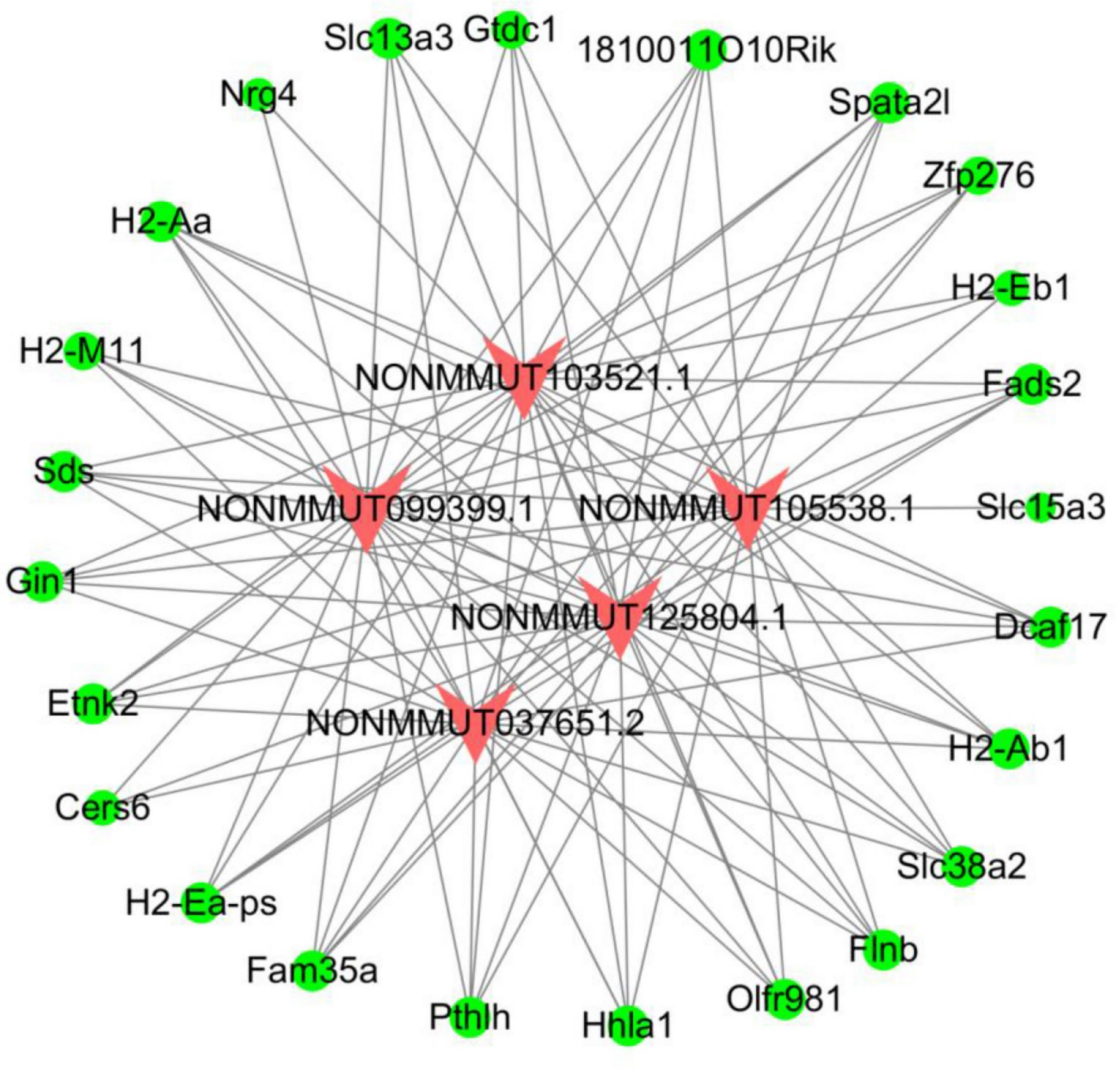
Co-expression network of the shared differentially expressed lncRNAs and differentially expressed mRNAs at eight time points in the liver of *Echinococcus multilocularis*-infected mice. The network was based on the Pearson correlation coefficient (|PCC value| > 0.9, *P* < 0.05). The red “V” denotes lncRNA while the green dot denotes mRNA.

To demonstrate the interactive relationship between the consistently altered DELs and DEMs, 31 DEMs and 68 DELs containing 2,108 relationships were estimated by calculating PCC values. According to the cutoff criteria (*P* < 0.05 and |PCC| > 0.9), the top five lncRNAs that had the most co-expression relationships with mRNAs were selected to set up the lncRNA-mRNA co-expression network (Fig 4). The following lncRNAs NONMMUT099399.1, NONMMUT105538.1, NONMMUT125804.1, NONMMUT103521.1 and NONMMUT037651.2 had 25, 23, 23, 23, 22 co-expression relationships with mRNAs, respectively. From the network, it was observed that NONMMUT099399.1 had the most relationships with DEM. Furthermore, 84% of the consistently altered DEMs (26 DEMs) had co-expressed relationships with these five lncRNAs, implying that these five lncRNAs most likely to have a regulatory role in the signaling pathways that displayed by KEGG analysis (Fig 3C).

### *Cis*-and *trans*-regulating target prediction of overlapped lncRNAs and construction of lncRNA-TF-mRNA network

**Fig 5:**
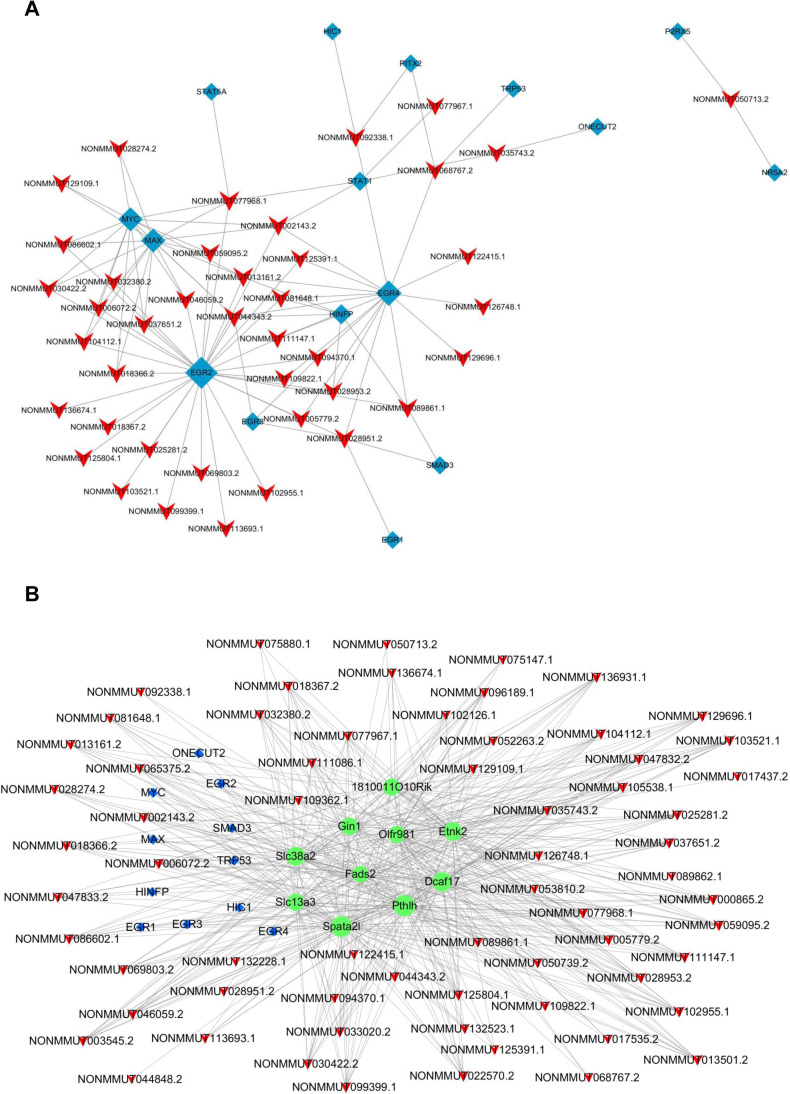
Regulating targets prediction of continuously dysregulated lncRNAs and construction of lncRNA-transcription factor-mRNA network. A. The network of transcription factors and continuously dysregulated lncRNAs, consisting of 16 transcription factors and correlated 41 lncRNAs. The red “V” denotes lncRNA, while the blue node denotes transcription factor. B. The lncRNA-transcription factor (TF)-mRNA network, consisting of 66 lncRNAs, 11 transcription factors and 10 correlated mRNAs (according to *p*-values). The red “V”, the blue node and the green node denote the lncRNA, the transcription factor and the mRNA, respectively.

Generally, lncRNAs perform functions by interacting with their targets, so we predicted the potential *cis*- and *trans*- target gene of these lncRNAs. We searched for protein-coding genes 10-kb up- and downstream of the 68 shared DELs. It was found that none of lncRNA was transcribed near (< 10 kb) the 31 DEMs neighbors. Then, the *trans*-regulatory functions of lncRNAs were predicted by evaluating the relationship between the TFs and lncRNA based on the cumulative hypergeometric test. We found that among the 68 consistently altered lncRNAs, 41 lncRNAs corresponded to 16 TFs (EGR1, EGR2, EGR3, EGR4, STAT1, STAT5A, SMAD3, MYC, HINFP, ONECUT2, P2RX5, NR5A2, TRP53, MAX, PITX2, and HIC1). These TFs could regulate the expression of these lncRNAs. In Fig 5A, among the 16 TFs, EGR2 had *trans*-regulating relationships with the most DELs. Besides, EGR1, EGR3, and EGR4 also had *trans*-regulating relationships with DELs, suggesting a possible regulatory relationship between the EGR transcription factor family and the host DELs in *Em* infection. Since each lncRNA-TF pair resulted from some genes enrichment, we selected the top 10 co-expressed mRNAs by *P*-value ranking to further set up a lncRNA-TF-mRNA ternary network (Fig 5B), which showed the potential regulatory relationships of the three.

### Validation of six upregulated DEMs involved in Toll-like and RIG-I-like receptor signaling pathways by qRT-PCR and WB

**Fig 6:**
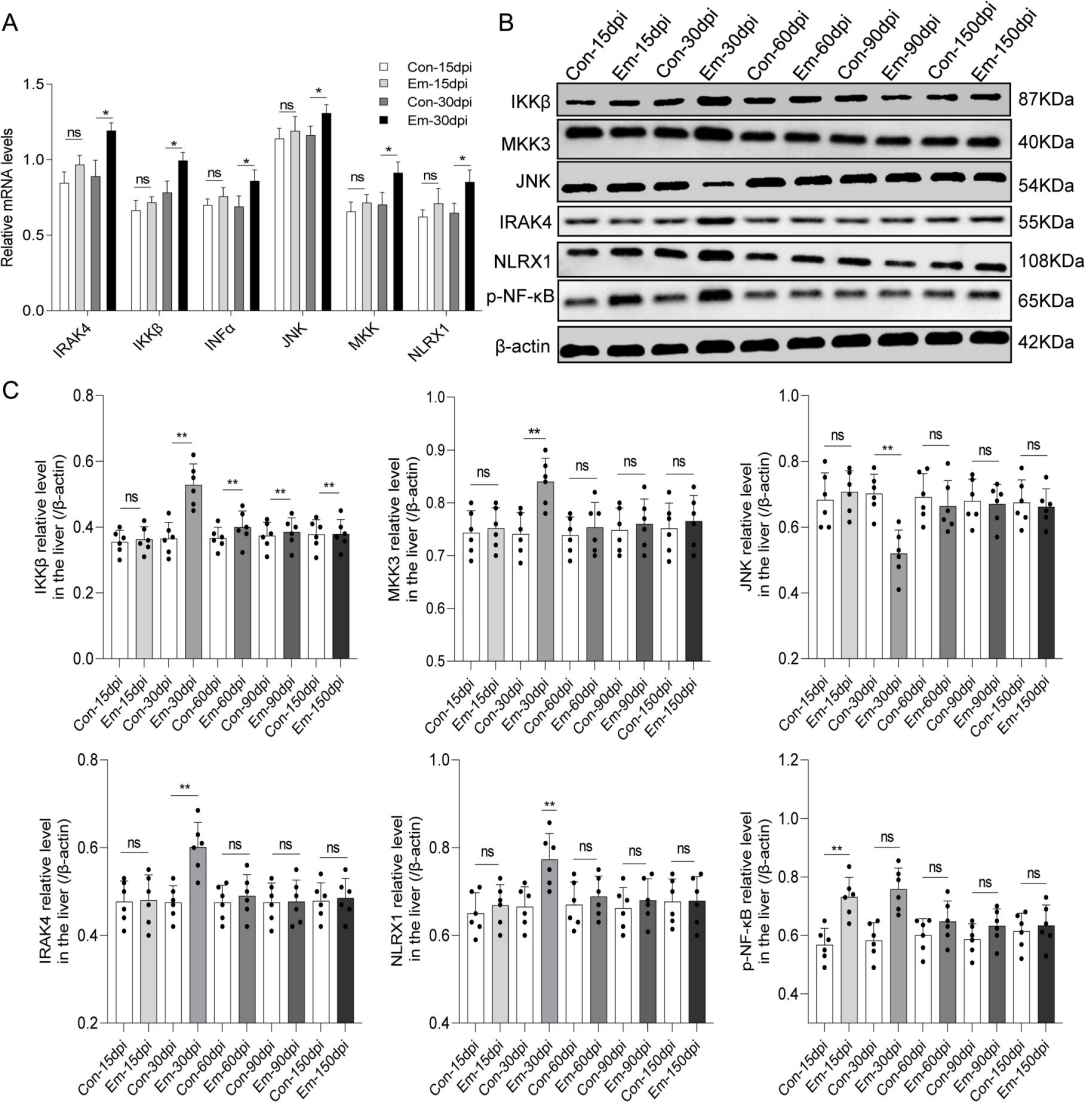
Validation of six up-regulated differentially expressed mRNAs involved in Toll-like receptor and RIG-I-like receptor signaling pathways. A. Quantitative real-time PCR (qRT-PCR) results of IRAK4, IKKβ, MKK, JNK, IFN-α, and NLRX1 in *Echinococcus multilocularis*-infected mouse liver at 15 and 30 days post-infection. The y-axis indicates the value of relative expression level (2^-⊿⊿Ct^) in Standard Deviation of the mean expression values. β-actin was an internal control. * *P* < 0.05 vs. corresponding control, **P < 0.01 vs. corresponding control, ns, no significance. B. Representative results of Western Blotting results of IRAK4, IKKβ, MKK, JNK, NLRX1, and *phospho*-NFκB in *Echinococcus multilocularis*-infected mouse liver at 15, 30, 60, 90, and 150 days post infection, with β-actin as a loading control. C. Quantitative results of protein expression of IRAK4, IKKβ, MKK, JNK, NLRX1, and *phospho*-NF-κB in *Echinococcus multilocularis*-infected mouse liver at 15, 30, 60, 90, and 150 days post-infection (*n* = 6). Significance was assessed using unpaired Student’s *t*-test, giving *P* values, *P < 0.05 vs. corresponding control, **P < 0.01 vs. corresponding control, ns, no significance.

Based on the KEGG analysis of DEMs, we investigated the influence of *Em* infection on Toll-like and RIG-I-like receptor signaling pathways of the host further. mRNA expression levels of six up-regulated DEMs (IRAK4, IKKβ, MKK, JNK, IFN-α, and NLRX1) were validated by qRT-PCR. Consistent with the microarray data in S10 Fig, the mRNA expression levels of IRAK4, IKKβ, MKK, JNK, IFN-α, and NLRX1 increased in the liver of CA mice compared to the control at 30 dpi (Fig 6A). The proteins expression of IKKβ, JNK, MKK, IRAK4, and NLRX1 at each time point were also determined. Compared with the Con groups, the expression of IKKβ, MKK, IRAK4, and NLRX1 proteins were promoted at 30 dpi. However, c-Jun amino-terminal kinase (JNK) decreased at 30 dpi (Fig 6B and 6C). Additionally, since nuclear factor Kappa B (NF-κB) is downstream of these two pathways, the protein expression of phospho-NF-κB (p-NF-κB) was tested. The result showed p-NF-κB significantly increased compared to the control at 15 and 30 dpi (Fig 6B and 6C). These results demonstrated that *Em* infection activated TLRs and LRLs signaling pathways at 30 dpi.

### Determination of cytokines and chemokines

**Fig 7:**
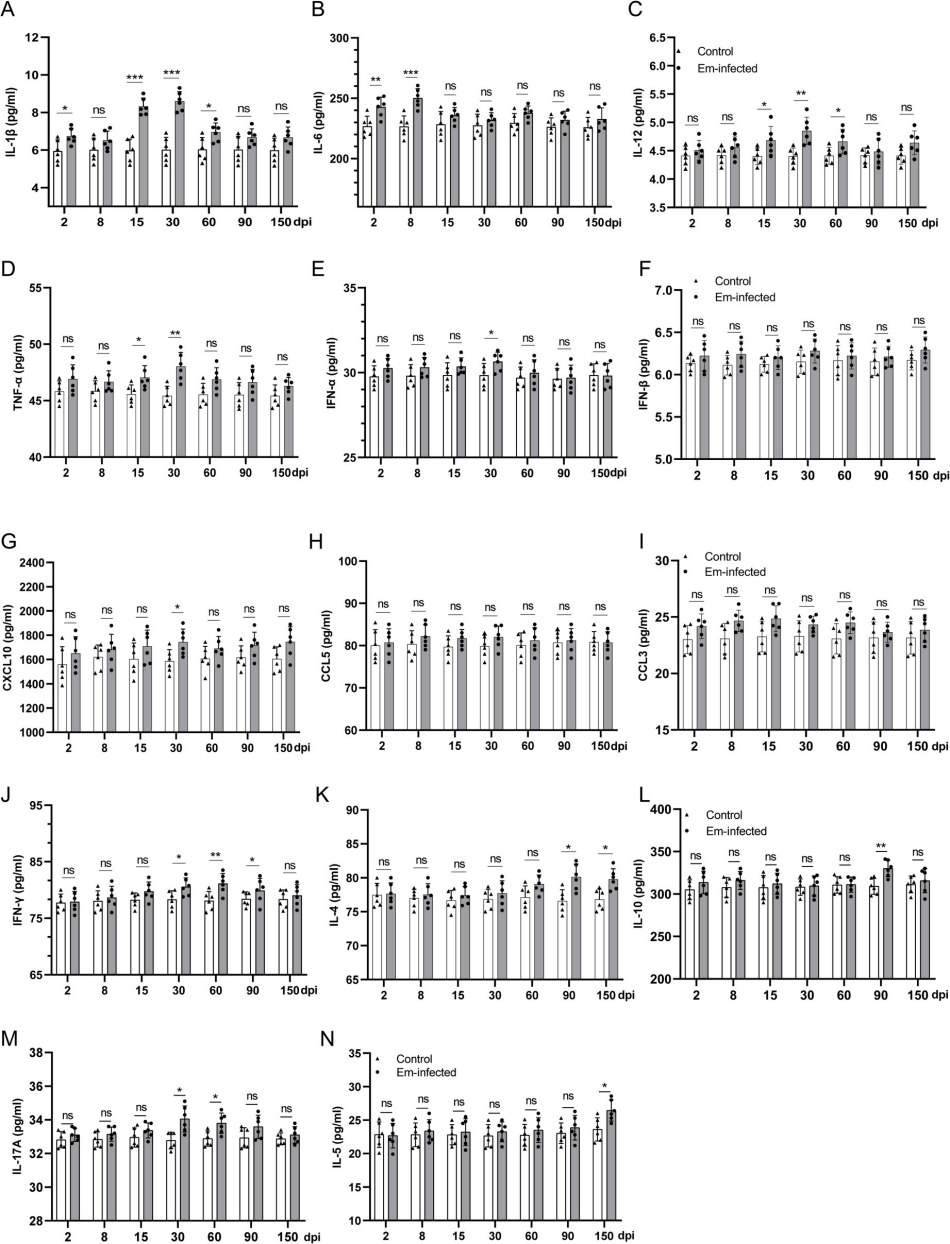
Assessment of cytokines/chemokines in the serum of *Echinococcus multilocularis*-infected mice. **A-H, J**. Levels of cytokines/chemokines in serum were measured by LEGENDplex Mouse Anti-Virus Response (13-plex) Panel including (A**)** IL-1β, (B) IL-6, (C) IL-12, (D) TNF-α, (E) IFN-α, (F) IFN-β, (G) CXCL10, (H) CCL5 and (J) IFN-γ. **I, K-N**. Levels of cytokines/chemokines in serum were measured by commercially available ELISA kits including (I) CCL3, (K) IL-4, (L) IL-10, (M) IL-17A and (N) IL-5 in *Echinococcus multilocularis*-infected mouse (*n*  =  6) at the indicated time points. Error bars represent the standard deviation (*n*  =  6). Significance was assessed using Student’s *t*-test, giving *P* values, * *P* < 0.05 vs. corresponding control, **P < 0.01 vs. corresponding control, ns, no significance.

To assess the impact of *Em* infection on the cytokines and chemokines induction downstream of TLRs and RLRs signaling pathway shown by KEGG database analyses (S7 and S8 Figs), the serum levels of IL-1β, IL-6, CCL5 (RANTES), TNF-a, IL-12, CCL3 (MIP1-a), IFN-a, IFN-β, and CXCL10 (IP10) at each time point were determined. As shown in Fig 7A–7I, the results showed that IL-1β, IL-12, TNF-α, IFN-α, and CXCL10 increased significantly at 30 dpi (*P* < 0.05). The level of IL-6 increased as early as 2 and 8 dpi and decreased at 30 dpi. The levels of IFN-β, CCL5, and CCL3 showed an increasing trend but the differences were not statistically significant compared to the control at 30 dpi (*P* > 0.05). Although not all the factors were significantly elevated, there was a clear underlying trend. Among these cytokines, as the most important proinflammatory cytokines, the serum levels of IL-1β and TNF-α increased markedly at 30 dpi, which may be related to increased level of p-NF-κB at 15 and 30 dpi, as represented in the WB results.

Additional, to assess the immunopathological effect of *Em* infection on the host, Th1-related cytokines IFN-γ, Th2-related cytokines IL-4, IL-5 and IL-10, and Th17-related cytokines IL-17A were determined at each time point. As shown in Fig 7J–7N, IFN-γ displayed a steadily increasing trend and peaked at 60 dpi (*P* < 0.01) before decreasing. IL-4 levels remained unchanged until 60 dpi and increased significantly at 90 and 150 dpi (*P* < 0.05). Compared to control, IL-10 and IL-5 were significantly higher at 90 and 150 dpi, respectively (*P* < 0.01, *P* < 0.05). IL-17A level increased significantly at 30 and 60 dpi compared with control following infection (*P* < 0.05, *P* < 0.05). These results suggested that *Em* induced Th1 and Th17-type cytokine (IFN-γ and IL-17A) from the beginning of the infection, while Th2-type cytokine (IL-4, IL-5 and IL-10) was induced at the late time points. During the whole infection time, IFN-γ played a role mainly before 90 dpi and anti-inflammatory factor IL-4, IL-5 and IL-10 were up-regulated at or after 90 dpi, indicating a response shift from Th1-orientation to Th2-orientation.

### Dynamic changes of Th1, Th2, and Th17-type CD4+ T-cells in the liver during *Em* infection course

**Fig 8:**
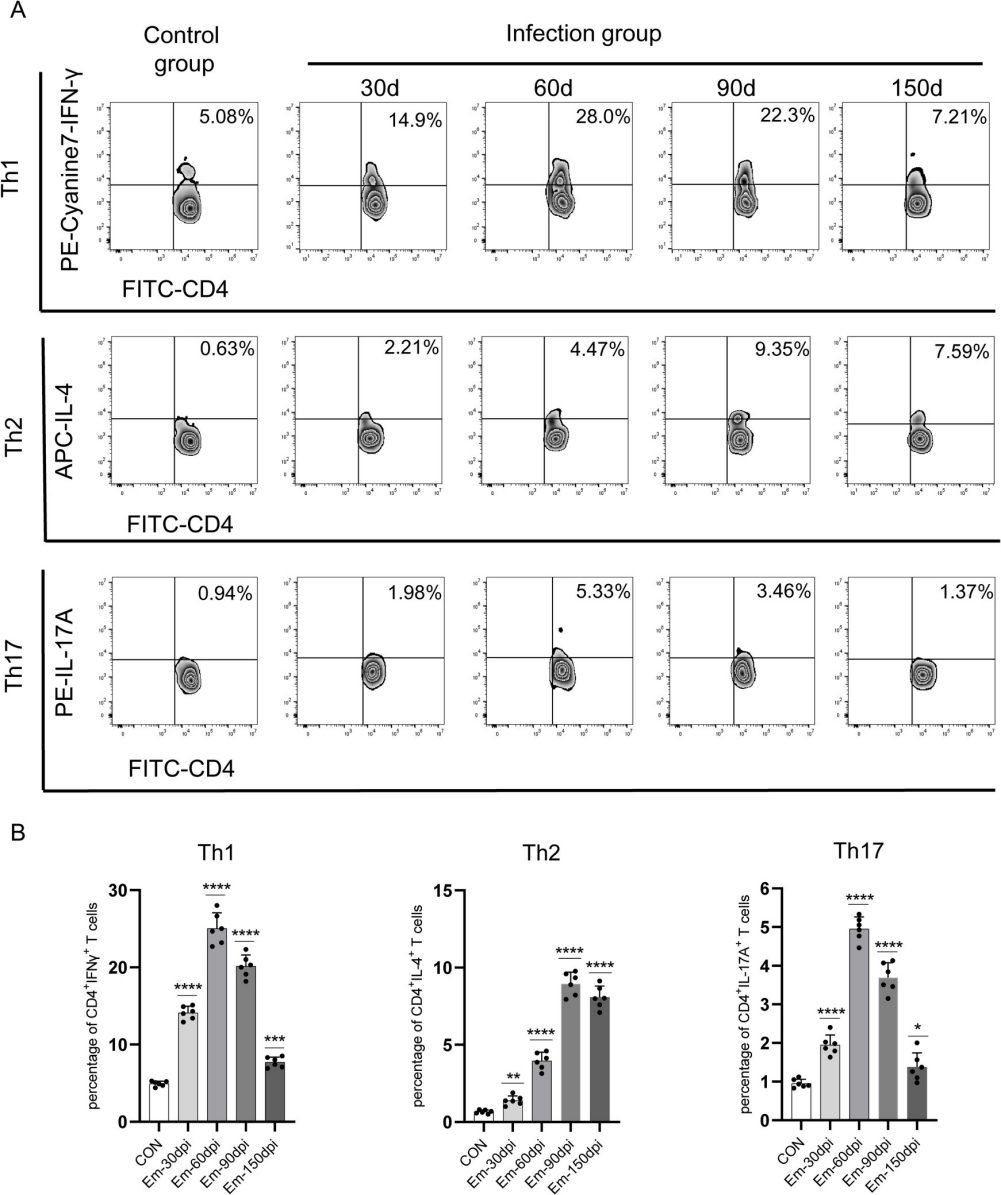
Dynamic changes of Th1, Th2, and Th17-type CD4^+^ T-cells in the liver of *Echinococcus multil*ocularis-infected mouse. A. Th1, Th2, and Th17-type CD4 ^+^ T-cells in the liver were determined by flow cytometric analysis at 30, 60, 90, and 150 days post-infection. The data are representative of three independent experiments (*n* = 6). B The percentages of Th1, Th2, and Th17-type CD4 ^+^ T-cells in the liver at 30, 60, 90, and 150 days post-infection (*n* = 6). Data are shown as the mean± SD. Significance was assessed using unpaired Student’s *t*-test, giving *p* values, *P < 0.05 vs. corresponding control, **P < 0.01 vs. corresponding control, ns, no significance.

To further assess the modulatory effect of *Em* on the local CD4^+^ T-cells, the percentage of Th1-type CD4^+^ T-cells (CD4^+^ IFN-γ^+^), Th2-type CD4^+^ T-cells (CD4^+^ IL4^+^) and Th17-type CD4^+^ T-cells (CD4^+^ IL-17A^+^) in the liver were assayed with flow cytometry at 2, 4, 8, 15, 30, 60, 90, and 150 dpi. The results showed there was no significant differences in the percentages of Th1, Th2 and Th17-type CD4^+^T-cells in the infected liver compared with the control at 2, 4, 8 and 15 dpi (S11 Fig). At 30, 60, 90, and 150 dpi, as shown in Fig 8A and 8B, as the infection ensued, the percentage of CD4^+^ IFN-γ^+^ cells was significantly higher than control at 30 dpi and reached a maximum at 60 dpi before decreasing; the CD4^+^ IL4^+^ cells percentage showed a gradual increasing and peaked at 90 dpi; the change of percentage of CD4^+^ IL-17A^+^ cells showed a similar trend to the percentage of CD4^+^ IFN-γ^+^ cells. These results suggested that Th1-type CD4^+^ T-cells (CD4^+^ IFN-γ^+^) were predominantly at the early infection stage, whereas Th2-type CD4^+^ T-cells (CD4^+^ IL4^+^) were induced significantly higher at the middle/late stage, suggesting an inflammatory Th1 response gradually converting into a mixed Th1/Th2 response later.

## Discussion

Human AE primarily occurs in the liver with decades-long period of pathologic changes leading to organ function failure. During the growth and proliferation of metacestode within the host liver, molecular interactions of host–parasite take place at every moment [28]. Therefore, elucidating the complex interplay of different biological macromolecules between host and parasite is critical for better understanding of host–pathogen relationships.

AE is characterized by a long asymptomatic period, the local interactions of host–pathogen before and at the early stages of cyst formation in human AE is poorly known. As rodents are the natural intermediate hosts in the conventional life cycle of *Em*, experimental mouse model of AE provides an important tool to investigate the interplay between the host and parasite at any stage of infection. According to the disease progression of human AE, there is a 3-stage course in the mice model of AE: early stage (before 60 dpi), middle stage (60 dpi to 180 dpi), and late stage (after 180 days) [29]. Given the vital regulatory roles of lncRNA in host–pathogen interactions, in this study, we used experimental mice model of secondary *Em* metacestode infection and selected eight different time points (2, 4, 8, 15, 30, 60, 90, and 150 dpi) to survey host lncRNAs and mRNAs profiles to capture a preliminary and comprehensive overview of gene expression changes during AE.

Our KEGG analysis results of DEMs at different infection stages corroborated previous findings [9,10]. Some pathways related to immune process, such as “immune response”, “inflammatory response”, and “antigen processing and presentation” were significantly enriched by the dysregulated mRNAs at the early stage (30 dpi). During the middle stage (90 dpi), the up-regulated DEMs were enriched on “transport”, “MAPK signaling pathway”, and “cell adhesion” pathways. And at nearly the late stage (150 dpi), the DEMs were associated to some metabolic pathways, such as “nitric oxide biosynthetic process”, “lipid metabolism”, and “central carbon metabolism”. Herein, we focused on the early stage of *Em* infection. In this study, the up-regulated genes at 30 dpi were significantly enriched in the TLRs and RLRs signaling pathways. This result was verified by the observed elevated levels of IL-1β (30 dpi), TNF-α (15 and 30 dpi), and IL-6 (2 and 8 dpi) in the sera, which requires the activation of pattern recognition receptor (PRR) to induce the inflammatory response. Studies had shown that TLR2 and TLR4 mRNA were significantly elevated in AE patients and TLR2 might play a role in regulating tissue infiltrative growth to facilitate the long-term survival of the parasite in the host [30,31]. Therefore, we measured the mRNA and protein expression levels of the up-regulated DEMs (IRAK4, IKKβ, MKK, JNK, and NLRX1) to investigate the effect of *Em* further. Increased mRNA and protein expression levels of IRAK4, IKKβ, MKK and NLRX1, and elevated serum levels of downstream cytokines/chemokines (IL-1β, IL-12, TNF-α, IFN-α, and CXCL10) demonstrated that the two pathways were activated by *Em* at 30 dpi.

In vitro experiment, high concentration of *Em* cyst fluid induced JNK-mRNA expression [32], which was consistent with the elevated JNK-mRNA level at 30 dpi in our results. Lin et al. reported up-regulated JNK-protein level during exposure to *Em* vesicle fluid or *Em* conditioned medium in vitro [33]. Zhang et al. found that activation of JNK was observed from 180 to 360 dpi in vivo experiments [29]. In our study, JNK protein expression level was reduced at 30 dpi. These observations suggested that protein level of JNK is dynamic during the long infection period and the different stages of *Em* infection. Components/secretions (*Em* vesicle fluid, *Em* conditioned medium or *Em* metacestodes) may have different modulatory effects on JNK expression/activation. We observed increased JNK-mRNA level at 30 dpi but decreased protein level. We postulated that JNK may be regulated at post-transcriptional/translational level by parasite components/secretions, which reduced JNK-protein level. It has been reported that EVs released by *Em* metacestodes can regulate the expression of cytokines and key components in the LPS/TLR4 pathway *in vitro* [34]. It is possible that some RNAs from *Em* interact with the JNK-mRNA, modulating the JNK-protein expression to attenuate JNK-mediated immune pathway activation such as RLRs pathway, which contributes to the parasite growth [35,36].

**Fig 9:**
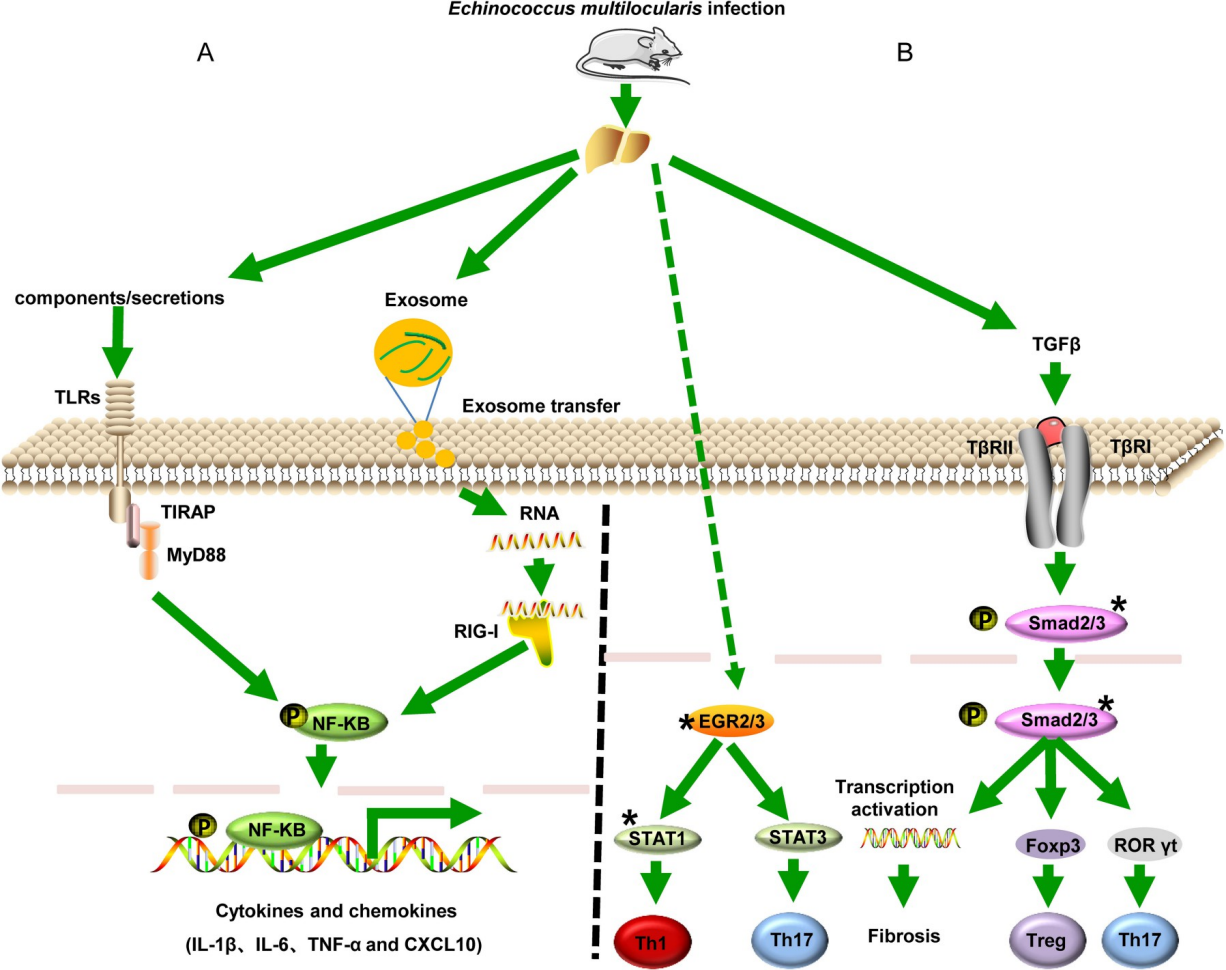
Schematic presentation of predicted targets for differentially expressed lncRNAs in *Echinococcus multilocularis*–host interplay (A) and the probable influence of *Echinococcus multilocularis* infection on the Toll-like receptor and RIG-I-like receptor signaling pathway of the host (B). “*” represents the predicted targets.

Overall, our results suggested that *Em* infection induced upregulation genes in RLRs or TLRs signaling pathway, then promoted NF-κB phosphorylation, which activated inflammatory cytokines expression, such as IL-1β, TNF-α and IL-12. What components/secretions of *Em* induce these responses and what are the specific mechanisms need to be further study (Fig 9A).

As the most prevalent and functionally diverse class of RNAs, lncRNAs play indispensable regulatory roles in epigenetics, transcription and post-transcriptional regulation by interacting with protein, DNA and RNA. The functions and range of lncRNA-mediated regulations in hosts following pathogen infection has received increased attention [37,38]. Compared with other studies of host mRNA profile after *Em* infection [9,10,29], our present study highlighted the possible roles of lncRNA by analyzing the co-expression relationships between DEMs and DELs. The function prediction analysis results of continuously dysregulated lncRNAs indicated that these lncRNAs may have modulatory roles in T helper 1 (Th1), T helper 2 (Th2) and T helper 17 (Th17) cell differentiation of the host. In response to *Em* infection, Th cells of the host can selectively differentiate into the Th1 or Th2 subset. Th1-oriented immunity is more likely to clear the parasite, whereas Th2-oriented immunity is more likely to develop chronic AE in patients [39]. Therefore, the differentiation of Th1 and/or Th2 cells and the Th1/Th2 imbalance are suggestive of a vital role in controlling the immunological response in AE. In addition, Th17 cells are another Th cell subtype that play a significant role in AE. It has been reported that Th17 cells are related to immunopathology of AE [40] and associated with the Th1/Th2-cell balance during *Em* infection [41,42]. The dynamic change of Th1, Th2 and Th17 response related cytokines levels and percentages of Th1, Th2 and Th17-type cells in the infected liver in the present study exhibited a similar trend, which indicated that Th1 and Th17-type CD4^+^ T-cells were predominant at the early infection stage whereas Th2-type CD4^+^ T-cells were significantly higher at the middle/late stage. These results confirmed changes in cell-mediated immunity in the host during the *Em* infection [43–45]. Our predicted results of potential regulatory roles of host lncRNAs in Th1, Th2 and Th17 cell differentiation provide additional useful clues for understanding the interaction of host and parasite.

The *trans-*regulating targets prediction analysis of these overlapped DELs revealed that a few TFs might be the interacting targets of DELs, including EGR genes, Trp53 (p53), Smad3, MAX, STAT1 and STAT5A. Notably, more than 20 DELs had *trans*-relationships with EGR2, which is reported to control Th17 cytokine expression and Th17 cells differentiation [46]. Th17 cells differentiation were also related to the T regulatory cell (Treg)/Th17 imbalance that were observed during the *Em* infection. Moreover, Tregs have the ability to affect antigen presentation and Th1-type immune responses suppression to favor *Em* metacestode survival [47]. It has also been shown that TGF-β/Smad pathway can regulate Treg/Th17 imbalance by driving the expansion of Foxp3^+^ Treg in the host [48–50]. Based on these findings, we hypothesized that these DELs may play an immunological modulate role by interacting with EGR2 and/or Smad3 to impact Th1, Th2 and/or Treg cells differentiation, hence facilitating parasite growth. Fig 9B provides a schematic illustration of the possible regulatory targets of these candidate DELs in *Em* infection. Investigating the interaction mechanism of these candidate lncRNAs with Smad3 and/or EGR2 to modulate the host Th cells subsets will be an exciting next step.

The present study has some limitations; to mention, more samples should be conducted in the microarray experiment, which requires us to invest more projects and funds. Moreover, our study was performed on liver samples without the spatial context of mRNA/lncRNA expression and valuable insights into the precise identification and respective location of DEMs and DELs should be further obtained by single-cell sequencing and spatial transcriptome sequencing. In what type of cells are these DELs/DEMs located, and whether such expression was limited to some immune cells in the liver or also present in hepatocytes. Such information would be helpful for exploration of regulatory mechanisms of lncRNA further.

Collectively, this study provided a new perspective to further understand the host–*Em* interplay in AE, and offered potential clues in understanding the influence of *Em* infection on host innate/adaptive immunity. Further studies are however needed concerning the regulatory detailed mechanism of lncRNA in the interactions between *Em* and the host, which will help in the future to design new antiparasitic strategies targeting the host’s non-protein-coding genome.

## Supporting information

S1 Text Protoscoleces preparation and *Echinococcus multilocularis* infected mouse model molecular identification. (DOCX)

S1 Table The details description of the mRNA and lncRNA probes and the gene composition of the Agilent Mouse lncRNA Microarray V3. (XLSX)

S2 Table PCR and qRT-PCR primers used in this study. (XLSX)

S3 Table Thirty-one differentially expressed mRNAs were found overlapped at all the time points by Venn analysis. (XLSX)

S4 Table Sixty-eight differentially expressed lncRNAs were found overlapped at all the time points by Venn analysis. (XLSX)

S1 Fig Confirmation of CA mice successfully infected by *Echinococcus multilocularis*. (A) The liver at each time point during the *Em* infection period. (B) PCR amplified *cox*1 fragments (Lanes 3–18). (C) PCR amplified *nad*1 fragments (Lanes 3–18). PCR products were examined in 1% (w/v) agarose gels stained with ethidium bromide. Lane 1, DL2000 molecular marker; Lane 2, positive controls.
(TIF)

S2 Fig Confirmation of collected liver samples without *Echinococcus multilocularis* contamination. **(**A) PCR amplified *cox*1 (Lanes 3–18) fragments. (B) PCR amplified *nad*1 (Lanes 3–18) fragments. PCR products were examined in 1% (w/v) agarose gels stained with ethidium bromide. Lane 1, DL2000 molecular marker; Lane 2, positive controls.
(TIF)

S3 Fig Volcano plot of differentially expressed lncRNAs in *Echinococcus multilocularis*-infected mice liver at 2 days post-injection (i), 4 days post-infection (ii), 8 days post-infection (iii), 15 days post-infection (iv), 30 days post-infection (v), 60 days post-infection (vi), 90 days post-infection (vii), 150 days post-infection (vii). The significantly up- and downregulated mRNAs are presented as red and blue dots, respectively (fold change > 2 and *P* < 0.05). The expression of mRNAs with | fold change | < 2 is presented as green dots (*P* < 0.05) and the expression of mRNAs not significantly differentially expressed is presented as gray dots (*P* > 0.05).
(TIF)

S4 Fig Volcano plot of differentially expressed mRNAs in *Echinococcus multilocularis*-infected mice liver at 2 days post-injection (i), 4 days post-infection (ii), 8 days post-infection (iii), 15 days post-infection (iv), 30 days post-infection (v), 60 days post-infection (vi), 90 days post-infection (vii), 150 days post-infection (viii). The significantly up- and downregulated mRNAs are presented as red and blue dots, respectively (fold change > 2 and *P* < 0.05). The expression of mRNAs with | fold change | < 2 is presented as green dots (*P* < 0.05) and the expression of mRNAs not significantly differentially expressed is presented as gray dots (*P* > 0.05).
(TIF)

S5 Fig Hierarchical clustering plot of differentially expressed lncRNAs and mRNAs in *Echinococcus multilocularis*-infected mice liver at indicated time points. (TIF)

S6 Fig Hierarchical clustering plot of differentially expressed mRNAs in *Echinococcus multilocularis*-infected mice liver at indicated time points. (TIF)

S7 Fig Toll-like receptor signaling pathway was enriched by up-regulated differentially expressed mRNAs in *Echinococcus multilocularis*-infected mice liver at 30 days post-infection by KEGG pathway analysis. The up-regulated genes are presented as red rectangles.
(TIF)

S8 Fig RIG-I-like receptor signaling pathway was enriched by up-regulated differentially expressed mRNAs (DEMs) in *Echinococcus multilocularis*-infected mice liver at 30 days post-infection by KEGG pathway analysis. The up-regulated genes are presented as red rectangles.
(TIF)

S9 Fig Ubiquitin-mediated proteolysis pathway enriched by downregulated differentially expressed mRNAs in *Echinococcus multilocularis*-infected mice liver at 15 days post-infection by KEGG pathway analysis. The downregulated genes are presented as green rectangles.
(TIF)

S10 Fig Expression levels of IRAK4, IKKβ, MKK, JNK, IFNα, and NLRX1 in *Echinococcus multilocularis*-infected mouse liver at 15 and 30 days post-infection from microarray data. The y-axis indicates the log_2_ FC by microarray. Error bars present as mean ± standard deviation (*n* = 4) at the indicated time point. Significance was assessed using Student’s *t*-test, giving *p*-values, * *P* < 0.05 vs. corresponding control, ** *P* < 0.01 vs. corresponding control, ns, no significance.
(TIF)

S11 Fig Dynamic changes of Th1, Th2, and Th17-type CD4^+^ T-cells in the liver of *Echinococcus multil*ocularis-infected mouse. (A) Th1, Th2, and Th17-type CD4 ^+^ T-cells in the liver were determined by flow cytometric analysis at 2, 4, 8, and 15 days post-infection. The data are representative of three independent experiments (*n* = 6). (B) The percentages of Th1, Th2, and Th17-type CD4 ^+^ T-cells in the liver at 2, 4, 8, and 15 days post-infection (*n* = 6). Data are shown as the mean± SD. Significance was assessed using unpaired Student’s *t*-test, giving *p* values, *P < 0.05 vs. corresponding control, **P < 0.01 vs. corresponding control, ns, no significance.
(TIF)
