# Supplementary material for: Understanding pathogen–host interplay by expression profiles of lncRNA and mRNA in the liver of Echinococcus multilocularis-infected mice
Source: PLoS Negl Trop Dis. 2022 May 31;16(5):e0010435. doi: 10.1371/journal.pntd.0010435 (PMC9187083; doi:10.1371/journal.pntd.0010435)
Supplement: S1 Text — (DOCX) [file pntd.0010435.s001.docx]

Supplementary document for methods

**Preparation of protoscoleces**

To isolate the protoscoleces, parasite tissue was first isolated from infected mice and homogenized [1]. The homogenate was subsequently filtered once through a 150-µm pore size nylon mesh, thus separating the protoscoleces from large pieces of metacestode tissue. The flow through was filtered through a 50-µm pore size nylon mesh, separating the protoscoleces from single cells and small cell clumps. They were then washed off the nylon mesh with sterile PBS and manually separated from equal sized vesicles using a pipette tip under a microscope.

***Echinococcus multilocularis* infected mouse model molecular identification**

*Echinococcus multilocularis* metacestodes DNA was extracted using DAeasy ® Blood ﹠Tissue kit (Qiagen, 69504). Molecular identification was undertaken by PCR-amplifying the complete fragments of the mitochondrial (mt) cytochrome c oridase subunit 1 (*cox*1) gene and NADH dehydrogenase subunit 1 (*nad*1) gene. The primers were designed [2] based on the multiple sequence alignment of *E. multilocularis* (its intermediate hosts include rodents) complete mt genomes [3] deposited in the National Center for Biotechnology Information (NCBI) GenBank (*nad*1-F: 5’-GAGTTTGCGTCTCGATGATAGG’-3, *nad*1-R: 5’-TCCCCAAAACCCACATTCTAC-3’; *cox*1-F: 5’-AGGTTTGACTTTCTCTTTGGTT-3’, *cox*1-R: 5’-CCAACAAATCCAAATAAACGG-3’). PCR products were purified directly from an agarose gel (1%) using an AxyPrep™ DNA Gel Extraction kit (AXVGEN, AP-GX-250G) and then sent to a commercial company (TsingKe Biotech, Xi’an, China) for sequencing. The sequences were submitted to NCBI GenBank, to search for homologous sequences using Basic Local Alignment Search Tools (BLAST), and then deposited in NCBI GenBank.


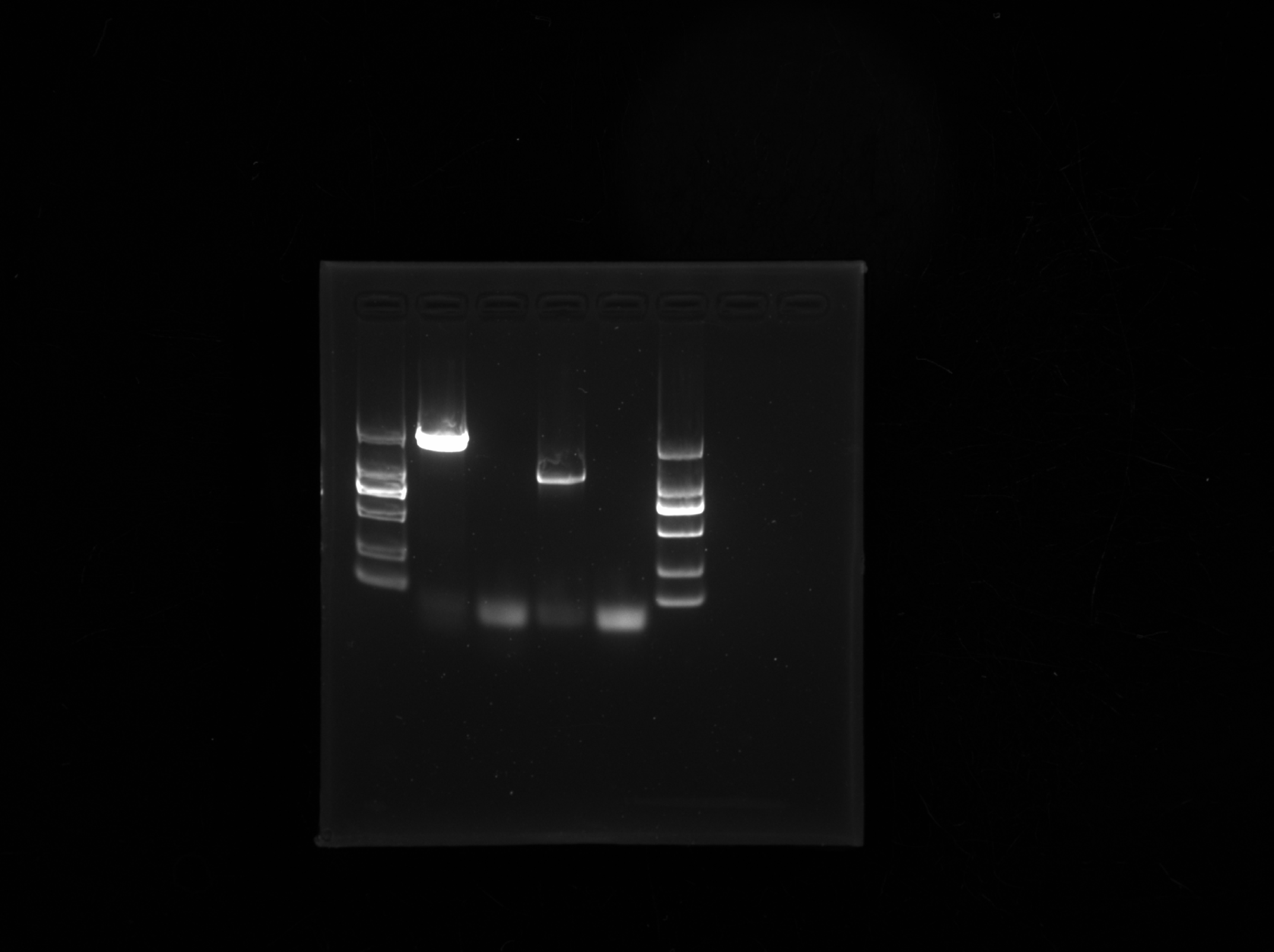


**2000**

**bp**

**1000**

**500**

**100**

**M**

**S**

**Con.**

**S**

**Con.**

**1**

**2**

**3**

**4**

**5**

**Figure legend：**PCR amplified *cox*1 (lane 2) and *nad*1 (lane 4) fragments examined in 1% (w/v) agarose gels stained with ethidium bromide: Lane 1, DL2000 molecular marker; Lane 2 and 4, *Echinococcus multilocularis* metacestodes, Lane 3 and 5, Controls.

**Reference**

1. Jura H, Bader A, Hartmann M, Maschek H, Frosch M. Hepatic tissue culture model for study of host-parasite interactions in alveolar echinococcosis. Infect Immun. 1996;64(9):3484-3490.
2. Li L, Chen B, Yan H, Zhao Y, Lou Z, Li J, et al. Three-dimensional hepatocyte culture system for the study of *Echinococcus multilocularis* larval development. PLoS Negl Trop Dis. 2018;12(3):e0006309.
3. Nakao M, Yokoyama N, Sako Y, Fukunaga M, Ito A. The complete mitochondrial DNA sequence of the cestode *Echinococcus multilocularis* (Cyclophyllidea: Taeniidae). Mitochondrion. 2002;1(6):497-509.
