# Supplementary figures and images for: Understanding pathogen–host interplay by expression profiles of lncRNA and mRNA in the liver of Echinococcus multilocularis-infected mice
Source: PLoS Negl Trop Dis. 2022 May 31;16(5):e0010435. doi: 10.1371/journal.pntd.0010435 (PMC9187083; doi:10.1371/journal.pntd.0010435)

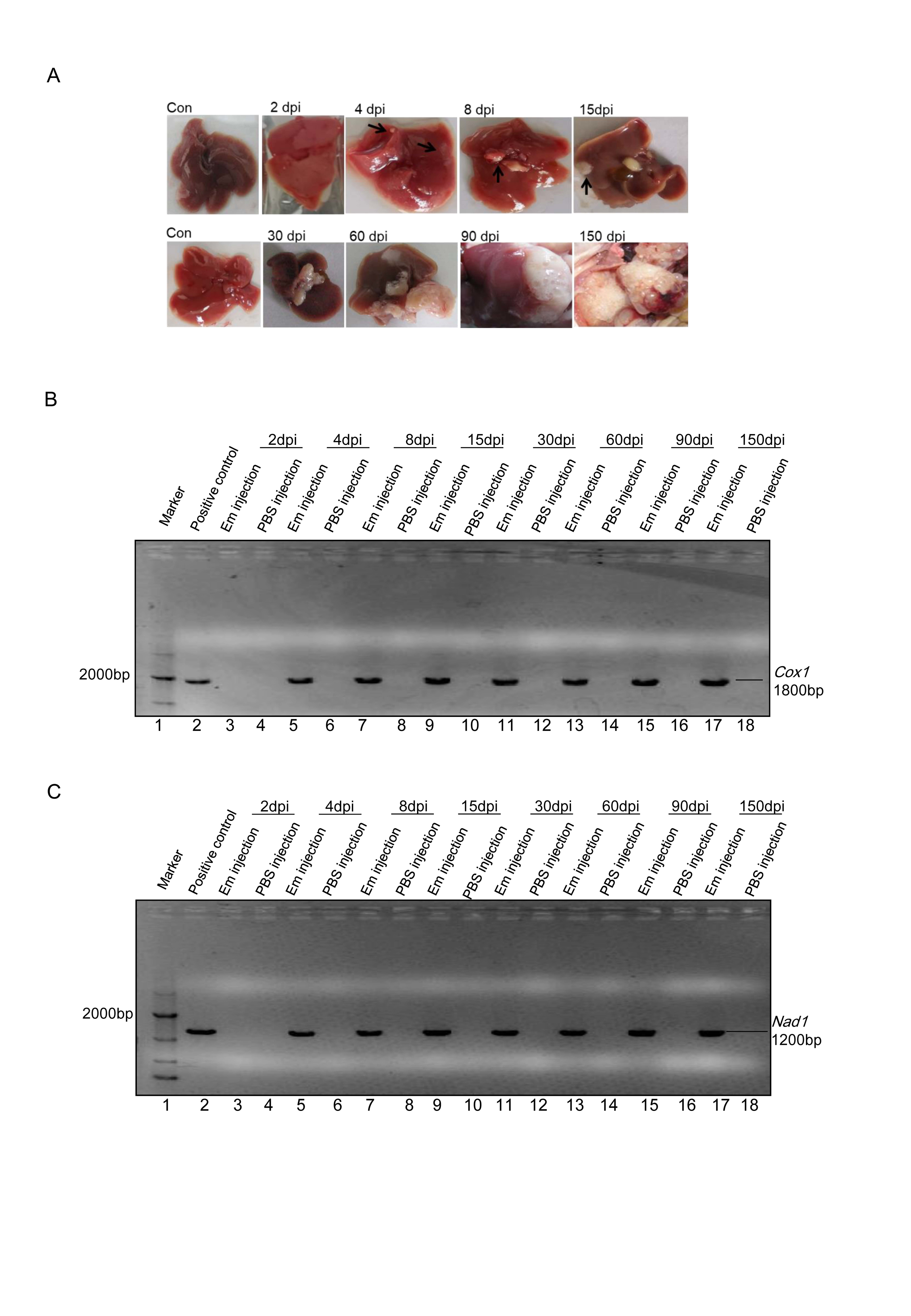

Supplement: S1 Fig — (A) The liver at each time point during the Em infection period. (B) PCR amplified cox1 fragments (Lanes 3–18). (C) PCR amplified nad1 fragments (Lanes 3–18). PCR products were examined in 1% (w/v) agarose gels stained with ethidium bromide. Lane 1, DL2000 molecular marker; Lane 2, positive controls. (TIF) [file pntd.0010435.s006.tif]

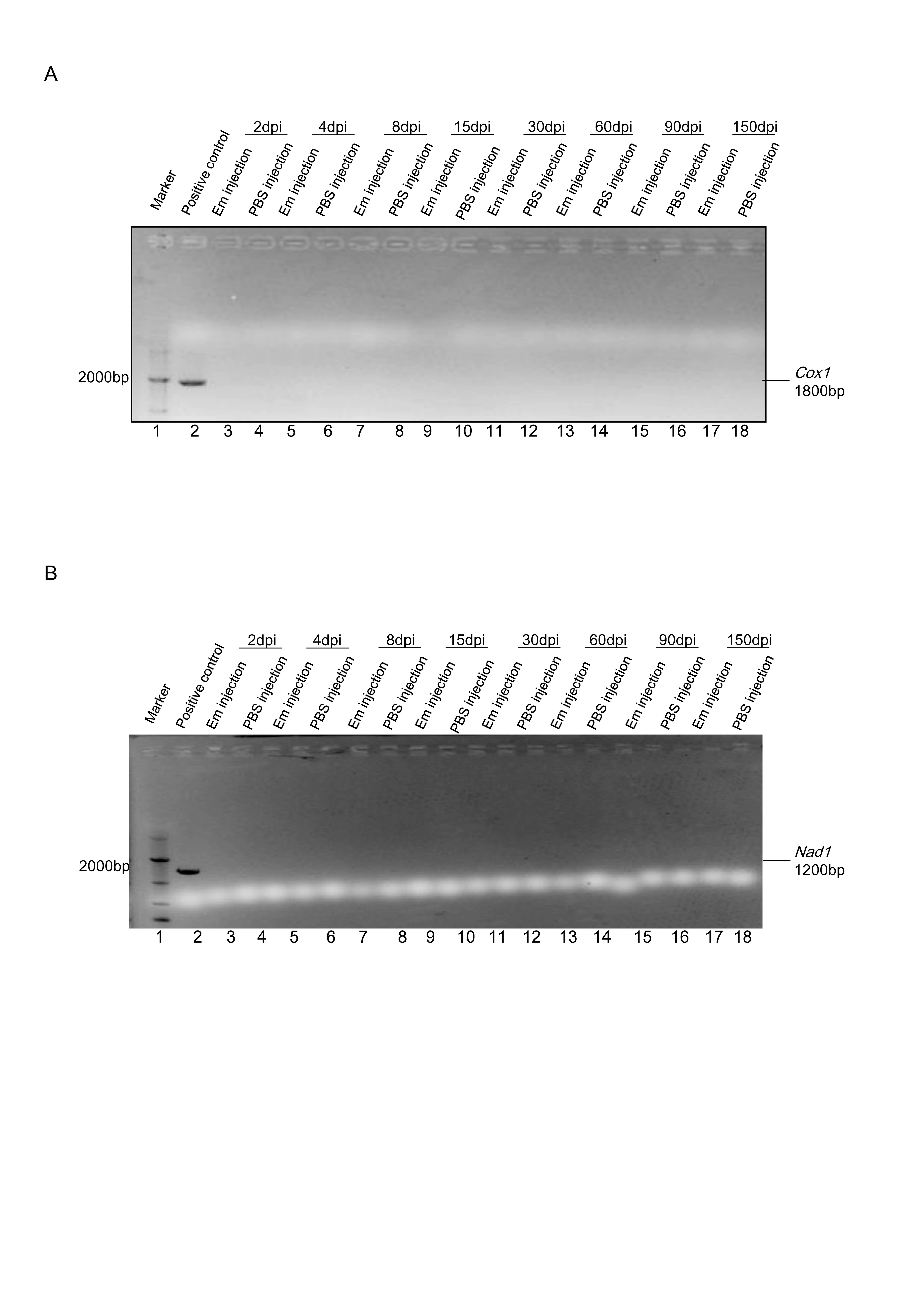

Supplement: S2 Fig — (A) PCR amplified cox1 (Lanes 3–18) fragments. (B) PCR amplified nad1 (Lanes 3–18) fragments. PCR products were examined in 1% (w/v) agarose gels stained with ethidium bromide. Lane 1, DL2000 molecular marker; Lane 2, positive controls. (TIF) [file pntd.0010435.s007.tif]

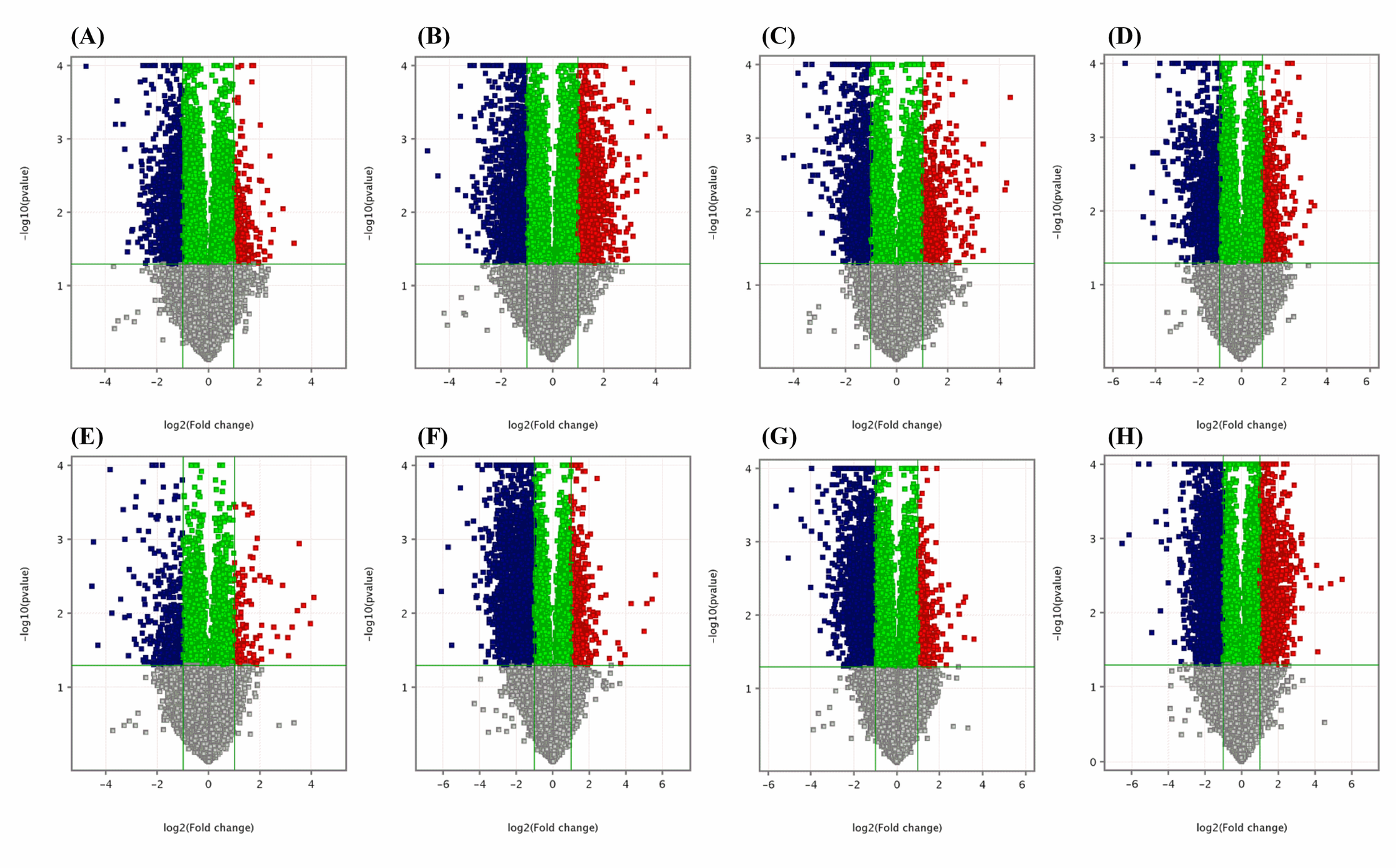

Supplement: S3 Fig — Volcano plot of differentially expressed lncRNAs in Echinococcus multilocularis-infected mice liver at 2 days post-injection (i), 4 days post-infection (ii), 8 days post-infection (iii), 15 days post-infection (iv), 30 days post-infection (v), 60 days post-infection (vi), 90 days post-infection (vii), 150 days post-infection (vii). The significantly up- and downregulated mRNAs are presented as red and blue dots, respectively (fold change > 2 and P < 0.05). The expression of mRNAs with | fold change | < 2 is presented as green dots (P < 0.05) and the expression of mRNAs not significantly differentially expressed is presented as gray dots (P > 0.05). (TIF) [file pntd.0010435.s008.tif]

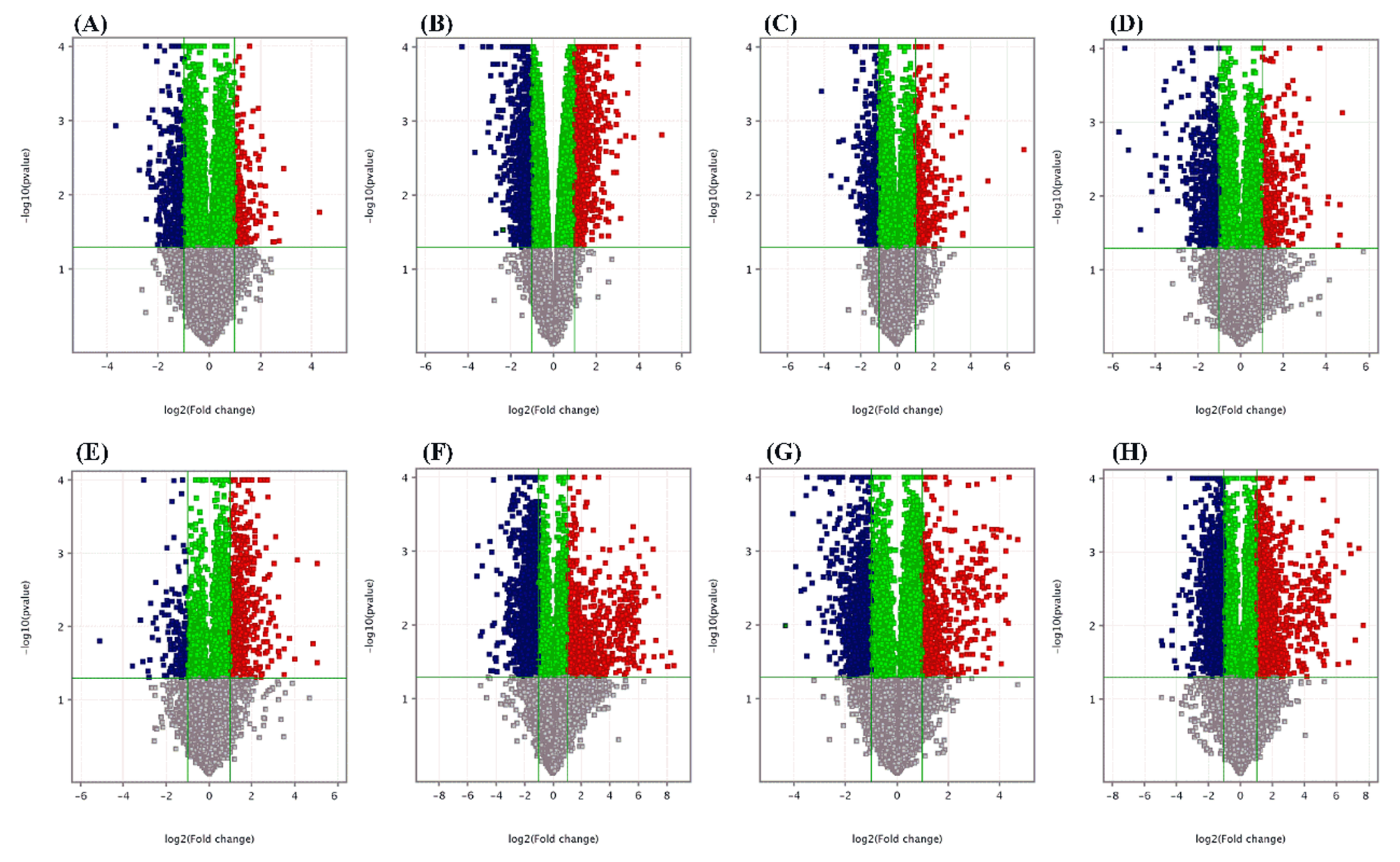

Supplement: S4 Fig — Volcano plot of differentially expressed mRNAs in Echinococcus multilocularis-infected mice liver at 2 days post-injection (i), 4 days post-infection (ii), 8 days post-infection (iii), 15 days post-infection (iv), 30 days post-infection (v), 60 days post-infection (vi), 90 days post-infection (vii), 150 days post-infection (viii). The significantly up- and downregulated mRNAs are presented as red and blue dots, respectively (fold change > 2 and P < 0.05). The expression of mRNAs with | fold change | < 2 is presented as green dots (P < 0.05) and the expression of mRNAs not significantly differentially expressed is presented as gray dots (P > 0.05). (TIF) [file pntd.0010435.s009.tif]

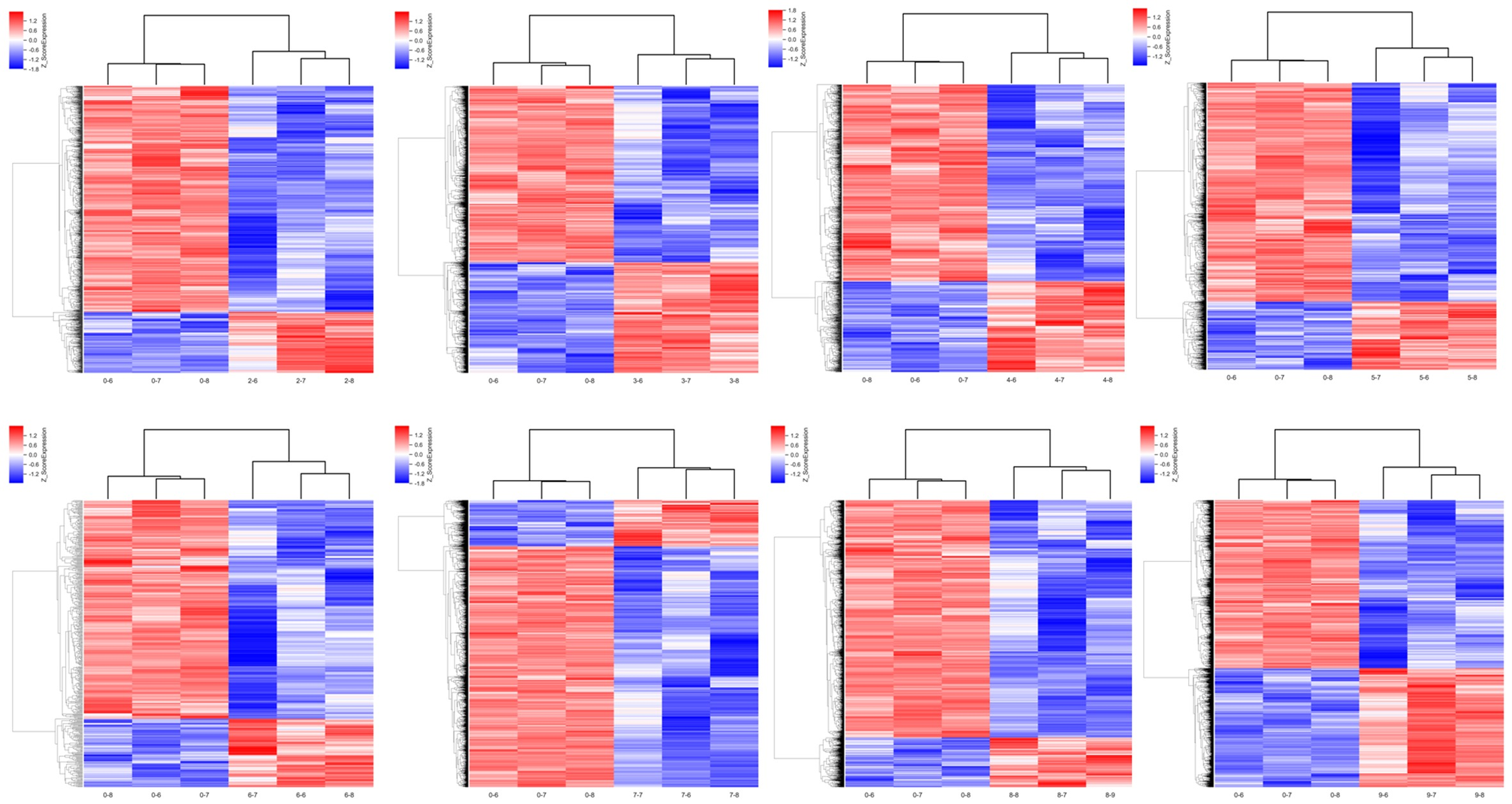

Supplement: S5 Fig — (TIF) [file pntd.0010435.s010.tif]

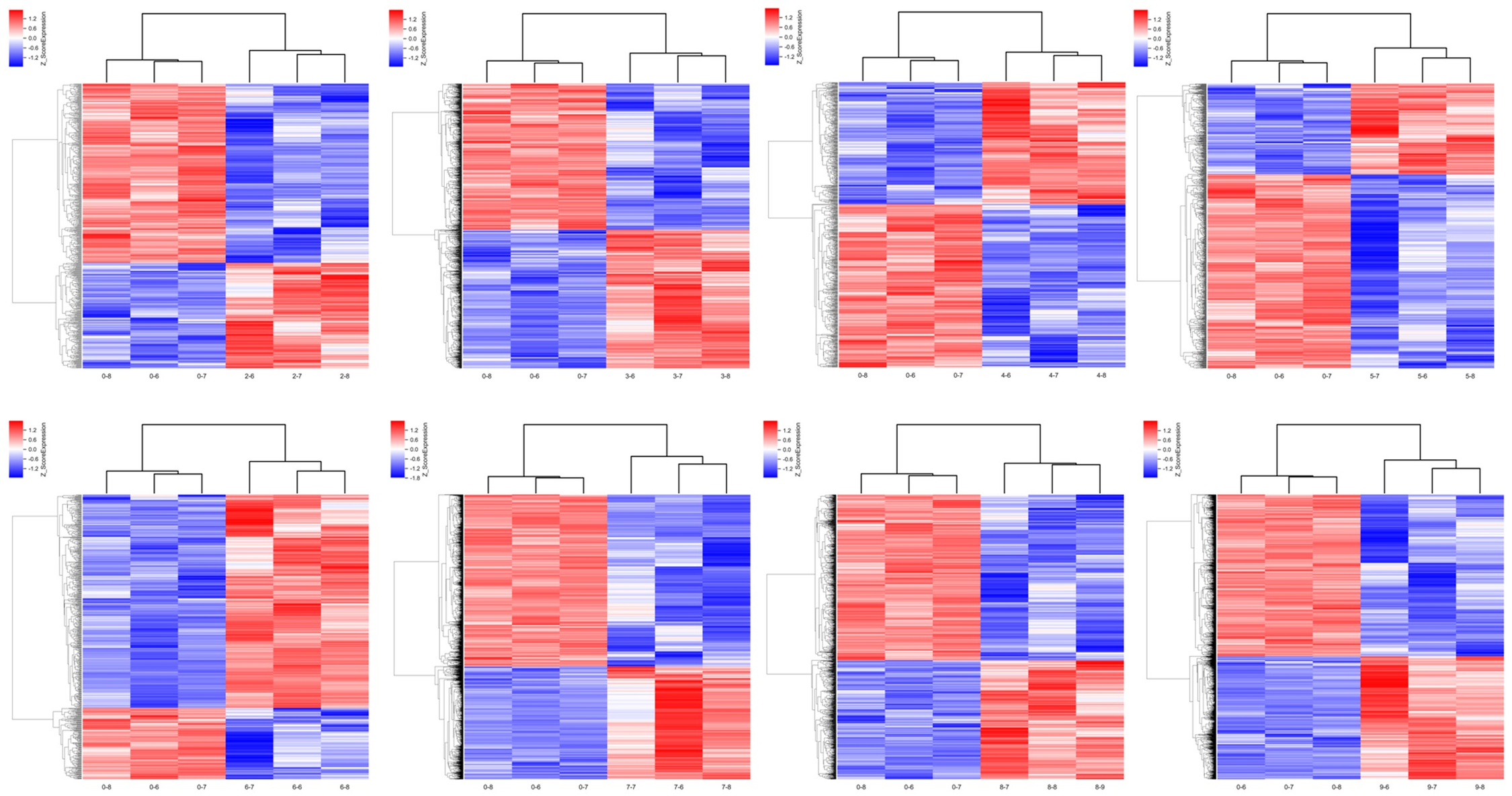

Supplement: S6 Fig — (TIF) [file pntd.0010435.s011.tif]

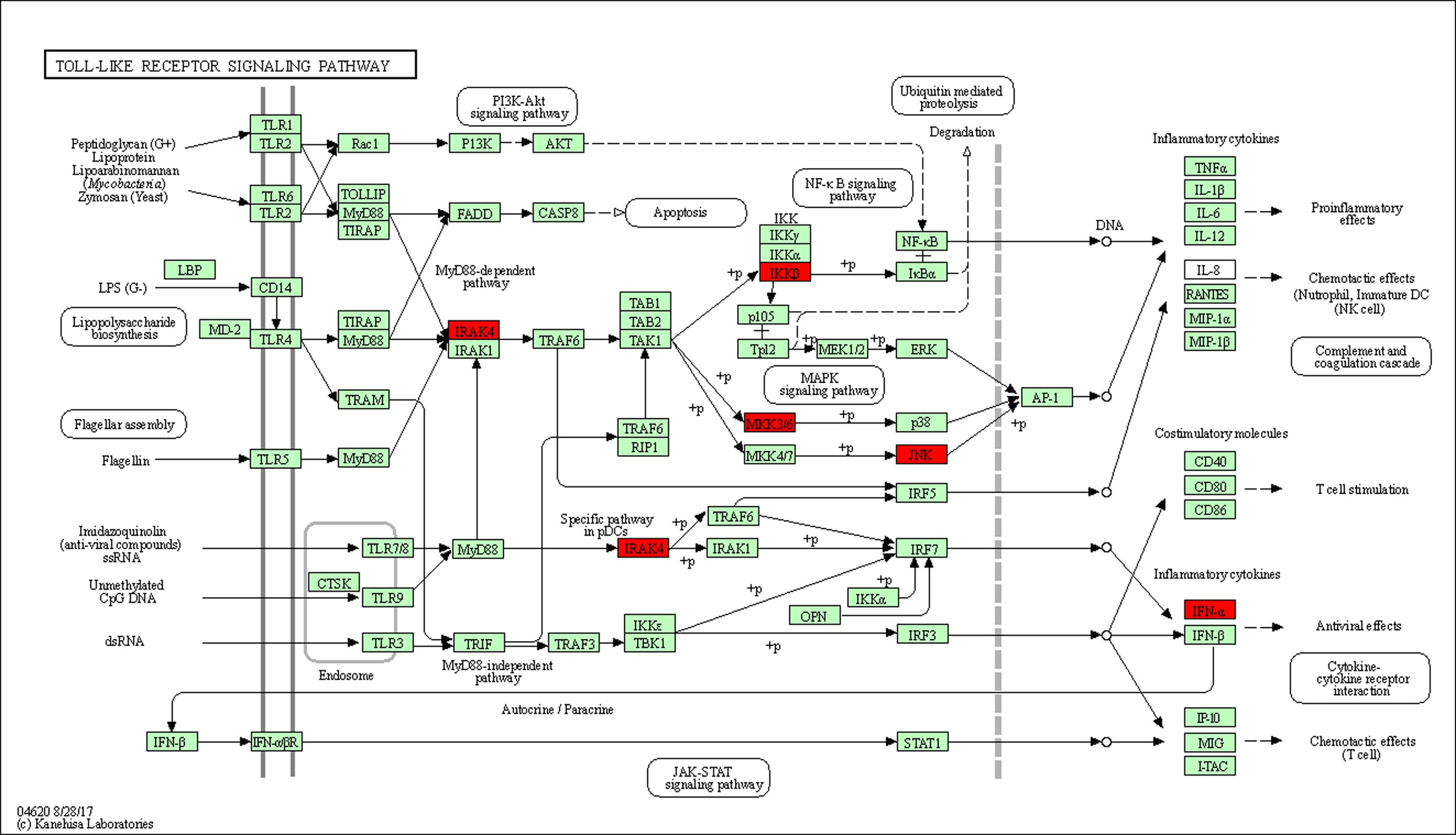

Supplement: S7 Fig — The up-regulated genes are presented as red rectangles. (TIF) [file pntd.0010435.s012.tif]

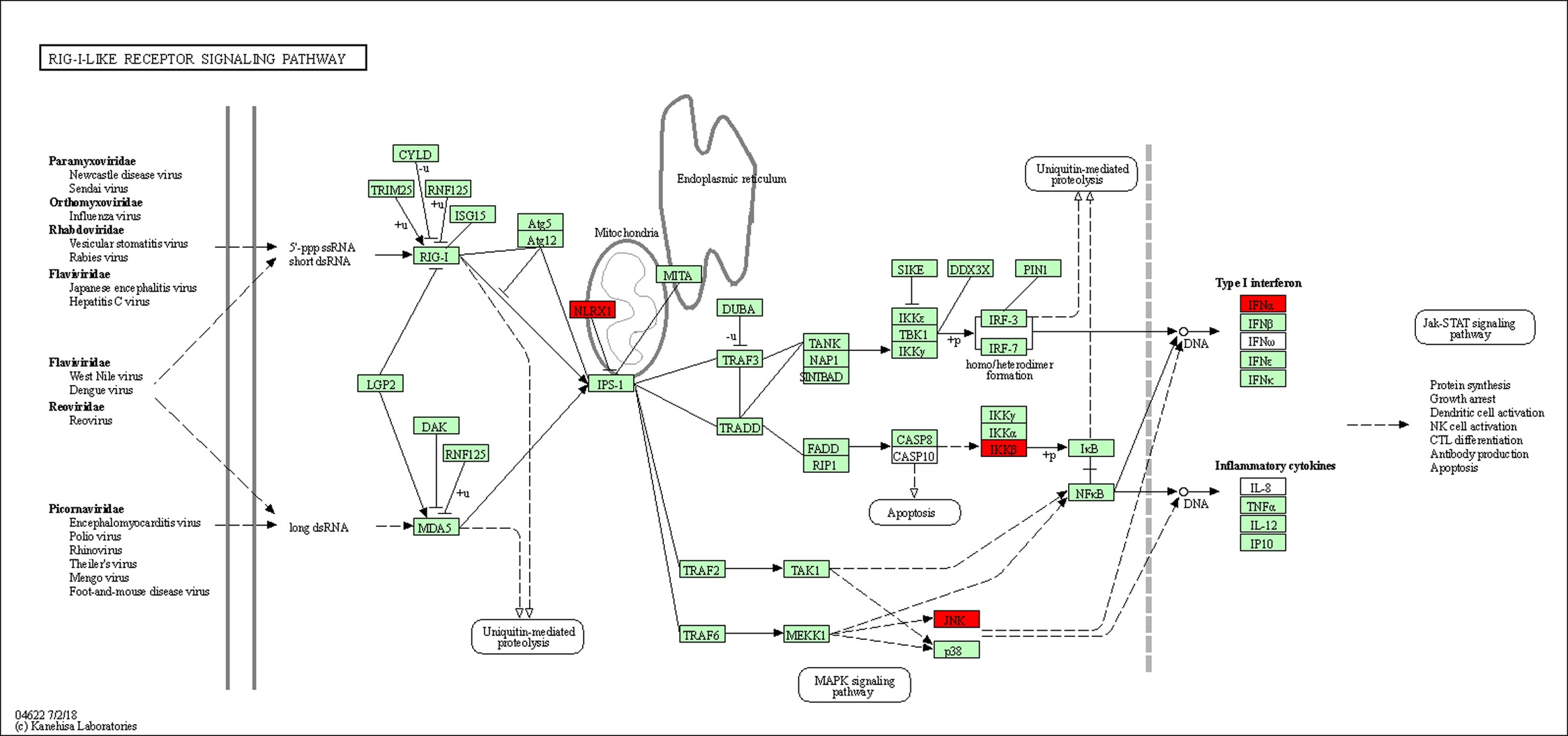

Supplement: S8 Fig — The up-regulated genes are presented as red rectangles. (TIF) [file pntd.0010435.s013.tif]

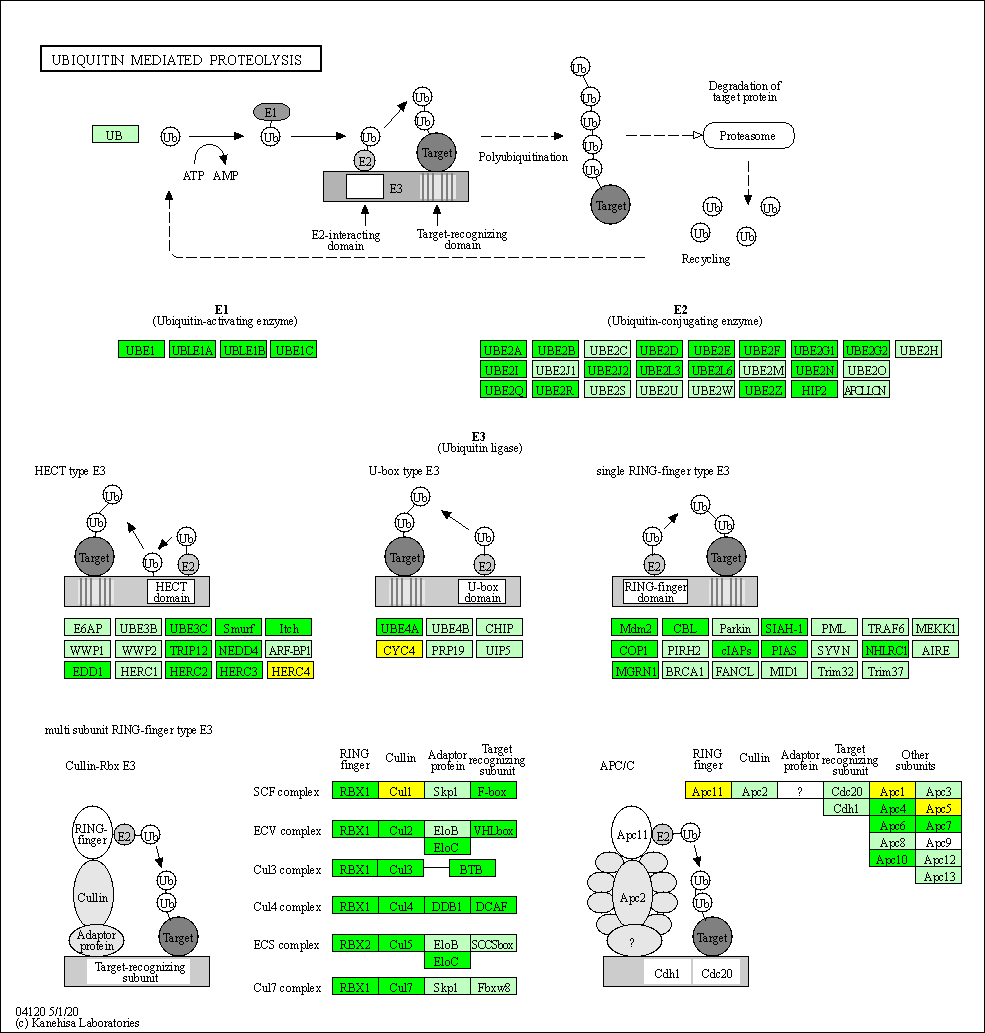

Supplement: S9 Fig — The downregulated genes are presented as green rectangles. (TIF) [file pntd.0010435.s014.tif]

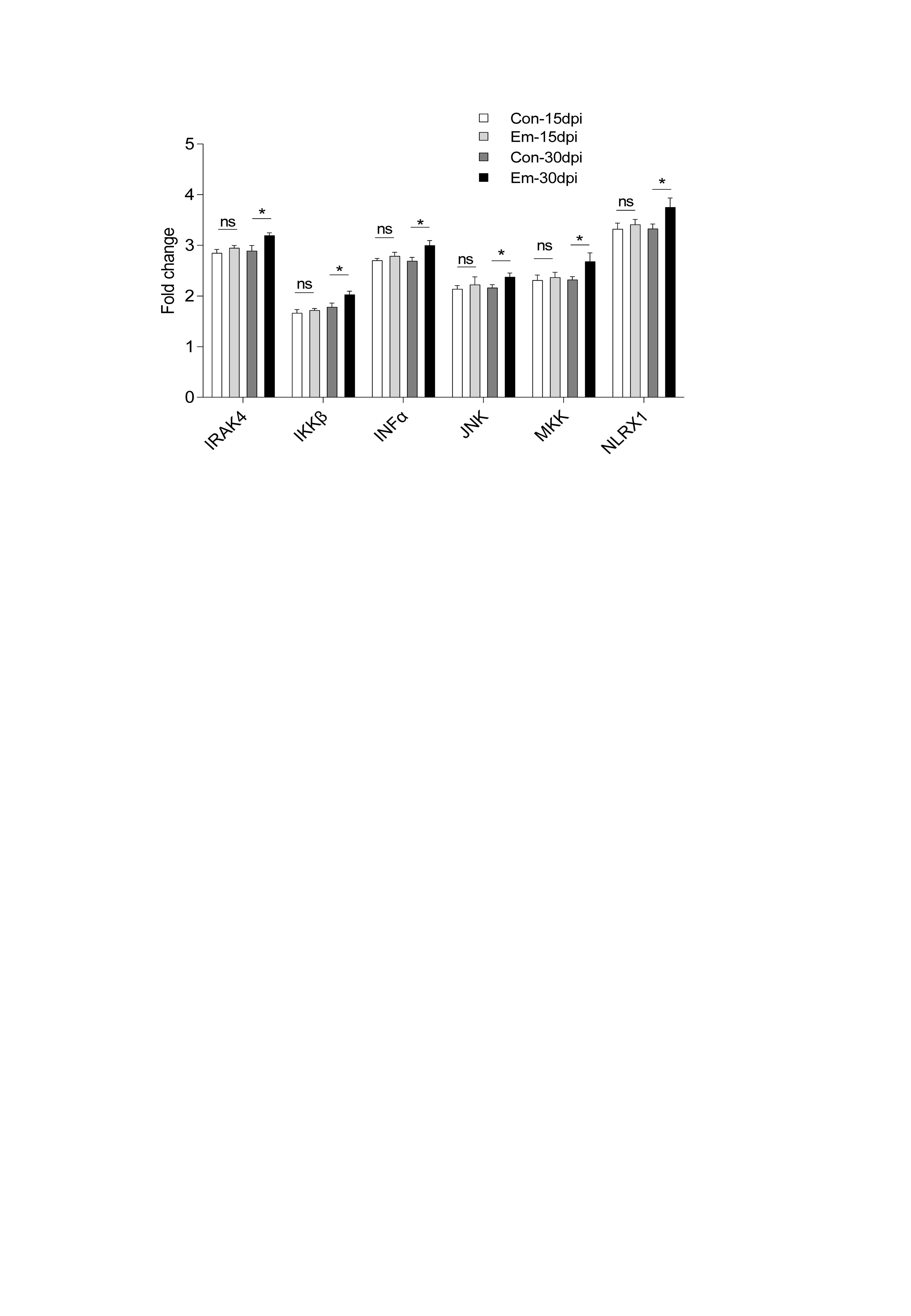

Supplement: S10 Fig — The y-axis indicates the log2 FC by microarray. Error bars present as mean ± standard deviation (n = 4) at the indicated time point. Significance was assessed using Student’s t-test, giving p-values, * P < 0.05 vs. corresponding control, ** P < 0.01 vs. corresponding control, ns, no significance. (TIF) [file pntd.0010435.s015.tif]

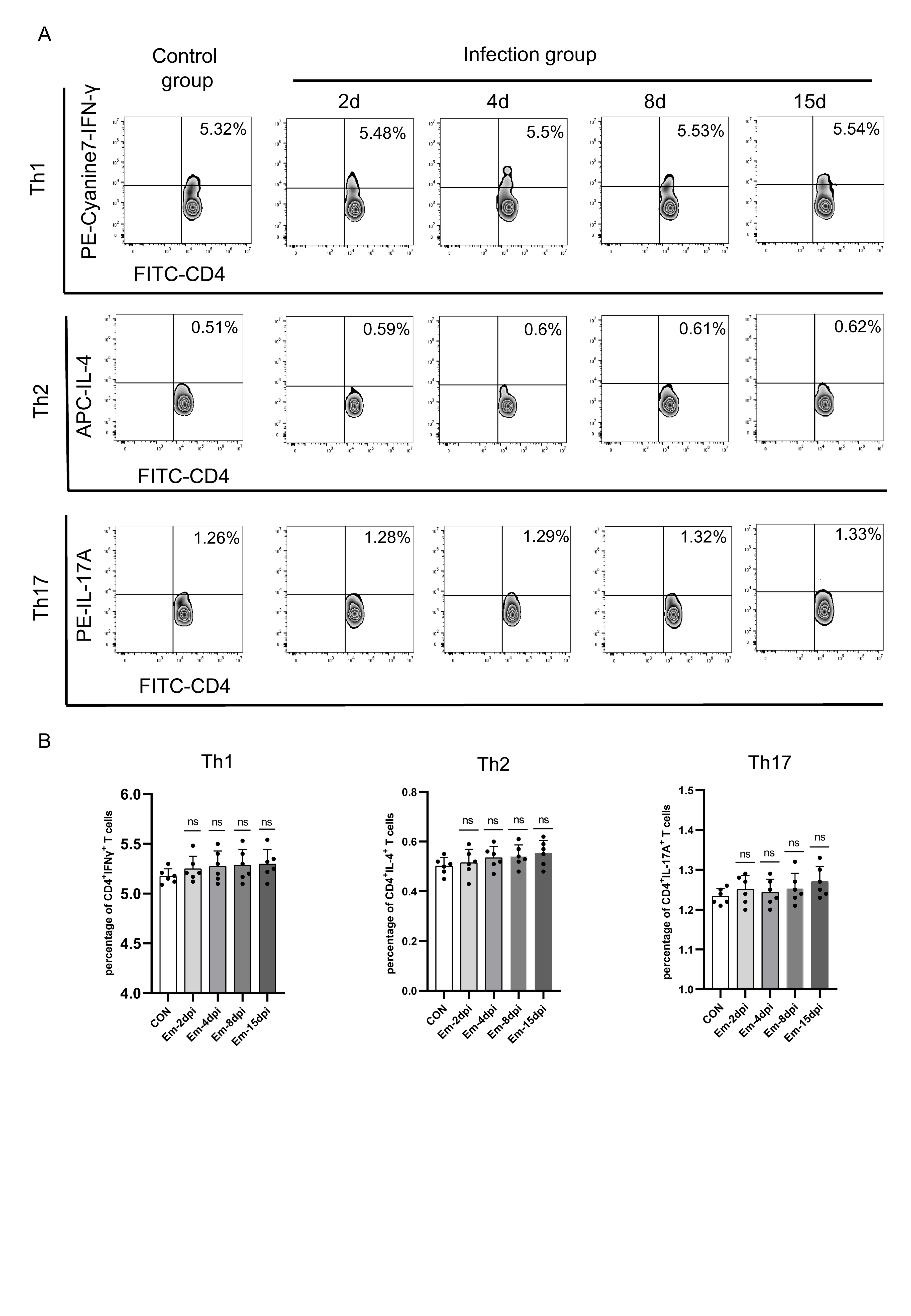

Supplement: S11 Fig — (A) Th1, Th2, and Th17-type CD4 + T-cells in the liver were determined by flow cytometric analysis at 2, 4, 8, and 15 days post-infection. The data are representative of three independent experiments (n = 6). (B) The percentages of Th1, Th2, and Th17-type CD4 + T-cells in the liver at 2, 4, 8, and 15 days post-infection (n = 6). Data are shown as the mean± SD. Significance was assessed using unpaired Student’s t-test, giving p values, *P < 0.05 vs. corresponding control, **P < 0.01 vs. corresponding control, ns, no significance. (TIF) [file pntd.0010435.s016.tif]
